# Image-Based Deep Learning for Cataract Diagnosis: Systematic Review and Meta-Analysis

**DOI:** 10.2196/78869

**Published:** 2026-04-29

**Authors:** Ruixi Li, Hongyi Li, Chong Li, Shuo Li, Linhong Lei, Dan Tao

**Affiliations:** 1 Department of Ophthalmology, The Second Affiliated Hospital of Kunming Medical University, Kunming, Yunnan Province, China; 2 Department of Ophthalmology, People's Hospital of Dongchuan District, Kunming, Yunnan Province, China; 3 Department of Ophthalmology, Kunming Children's Hospital (The affiliated Children's Hospital of Kunming Medical University), Kunming, China; 4 Department of Ophthalmology, Qilin District People's Hospital of Qujing City, Qujing, Yunnan Province, China; 5 Department of Ophthalmology, Yunnan Provincial Maternal and Child Health Hospital, No. 200 Gulou Road, Wuhua District, Kunming, Yunnan Province, 650051, China, +86 13987610751

**Keywords:** image-based, deep learning, meta-analysis, cataract, systematic review

## Abstract

**Background:**

Cataracts are an eye condition characterized by high prevalence and blindness-inducing potential, and effective approaches are required for their early diagnosis, underscoring the clinical significance of this study.

**Objective:**

This study aims to evaluate the performance of deep learning (DL) in cataract diagnosis and assess its potential as an effective tool for automated diagnosis, and compare the diagnostic accuracy of DL versus both machine learning and human experts.

**Methods:**

A systematic search was conducted in Web of Science, Embase, IEEE Xplore, PubMed, and Cochrane Library until April 1, 2025, for studies on image-based DL for cataract detection or clinical subtype classification. The included studies were assessed for the risk of bias (RoB) using Quality Assessment of Diagnostic Accuracy Studies-2 (QUADAS-2). Bivariate mixed effects models were used for data analyses, and publication bias was assessed by Deeks’ funnel plots.

**Results:**

Sixty-three studies were finally included. The quality assessment indicated a high or unclear RoB in the patient selection (34 studies) and index test (44 studies) domains. Meanwhile, in the reference standard domain, the risk of bias was high or unclear in only 2 studies. Image-based DL achieved a sensitivity of 96% (95% CI 0.95‐0.97) and a specificity of 98% (0.96‐0.98) for cataract detection, with an area under the ROC curve (AUC) of 0.99 (0.98‐1.00). For cataract classification, the sensitivity and specificity of image-based DL were 94% (0.93‐0.96) and 97% (0.96‐0.98), respectively, with an AUC of 0.99 (0.98‐0.99). Despite the strong overall performance, the model’s generalization capability was challenged by its lower performance observed on independent external datasets (detection: sensitivity 87%, specificity 93%; classification: sensitivity 89%, specificity 90%), potentially attributable to domain shift between the training and validation data.

**Conclusions:**

Image-based DL has demonstrated high precision in the detection and classification of cataracts, showing potential advantages over traditional machine learning methods, though validation remains limited. Its performance falls within the range of reported accuracy of human experts, highlighting the high feasibility of automated diagnosis. However, validation data limitations, coupled with moderate-quality evidence and high heterogeneity, constrain the utility of DL in auxiliary diagnosis. The model’s sensitivity dropped to 87% in external validation, restricting its generalization capability, so caution should be exercised in broad clinical implementation.

## Introduction

The lens is a biconvex, transparent structure in the anterior segment of the eye, which focuses light to project images of objects at varying distances onto the retina. A cataract, defined as clouding of the lens, is primarily an age-related degenerative disease, and congenital and pediatric cataracts also occur [1]. Early cataracts are asymptomatic, but progressive clouding can lead to visual impairment, greatly reducing quality of life and productivity [2]. Cataracts are the major cause of visual loss worldwide. In 2020, the estimated number of the blind aged 50 years and older was 15 million, and moderate to severe visual disorders due to cataracts affected an estimated 79 million people aged 50 years and older [2]. These figures suggest the number of patients with blindness and moderate to severe visual disorders rose by 30% and 93%, respectively, versus the year 2000 [3]. Blinding cataracts refer to cataracts causing severe visual impairment (visual acuity ≤3/60) or blindness (visual acuity <3/60) according to the WHO (World Health Organization) and *ICD-11* (*International Classification of Diseases, 11th Revision)* [4]. About 94% of blinding cataracts occur in low- and middle-income countries (LMICs), and cataract-related visual disorders are causally related to poverty in low-resource settings (LRS) [1]. As the conventional diagnosis of cataracts relies on ophthalmologists and complex equipment, it is subjective and requires health care resources, making it difficult to satisfy the need for large-scale screening. Moreover, due to the limitations of infrastructure and the lack of trained personnel in LMICs, inequalities are present in early cataract detection and severity classification.

Deep learning (DL) can process pixel-level information that cannot be recognized by the human eye to greatly contribute to the analysis of medical images, assist doctors in clinical decision-making, and enhance screening efficiency. As a branch of artificial intelligence (AI), DL models are inspired by the brain and specialized in pattern recognition [5]. Therefore, DL has been rapidly evolving with broad prospects in the field of medical image analysis involving disease detection, classification, segmentation, and image registration [6]. For example, convolutional neural networks (CNNs), as the primary technique of DL for image learning, perform excellently in image classification and feature extraction, making it a cornerstone in medical imaging [7]. Residual network (ResNet), one of the landmark architectures of CNNs, has contributed to the development of DL, especially with outstanding performance in benchmarking in image recognition and classification [8]. The superiority of the ResNet model in the medical field lies in its capability to efficiently train the deep network and raise the accuracy of image recognition [9].

The strategic importance of AI is to raise the quality of care and potentially reduce costs in high-income economies and to address critical health care issues and staffing shortages and provide access to specialized skills in LMICs [10]. Nowadays, DL is developed mostly based on data from high-income countries and regions and relies on high-resolution images and advanced electronic devices. In remote regions, however, fragmented health care systems are generally characterized by insufficient infrastructure, a shortage of professionals, and a lack of health care resources, so it is difficult to guarantee the quality of screening and diagnosis. The following problems are present in the available DL models: (1) lack of compatibility: mismatch with the low-cost equipment used in LRS [10]; (2) lack of generalization capability: significant decline in the model performance in real-world scenarios, especially under different lighting conditions or in different patient populations [11]; (3) lack of clinical validation: a recent systematic review of studies assessing the use of AI algorithms for medical image analysis found that only 6% of the included studies (n=516) conducted external validation, and the assessment of the diagnostic efficiency was lacking [12]; and (4) training data bias and lack of diversity in data (race, age, and disease subtypes) weaken the model’s generalization capability [13].

In the past 5 years, breakthroughs have been made in the use of DL for ophthalmic image analysis in diabetic retinopathy (DR), retinopathy of prematurity, and glaucoma. By automating the massive processing of ophthalmic images, DL can achieve a more accurate and rapid diagnosis of cataracts, reducing the doctors’ subjectivity and errors of conventional methods. Moreover, DL can fuse multiple image data (eg, slit lamp, fundus, and optical coherence tomography [OCT] images) to make a comprehensive and accurate diagnosis through multimodal image analysis. In addition, diagnostic results can be obtained quickly from the DL model with real-time diagnostic capability, greatly improving work efficiency and achieving large-scale screening and early diagnosis, especially in primary health care institutions and LRS [14]. Many DL-based diagnostic tools have been approved by the US Food and Drug Administration (FDA), but they require further evaluation and independent quality review [15]. For example, IDx-DR [16,17], the first AI system approved by the FDA, is a CNN-based system for automated screening for DR, with high sensitivity and specificity, which can contribute to the early diagnosis and lower the risk of visual loss in clinical practice, especially in LRS.

Image-based DL has exhibited greater potential for automatic cataract detection and classification using fundus and slit lamp images. However, the existing findings are still heterogeneous, and a systematic review of DL algorithms for cataract image analysis is still lacking. Therefore, this study was conducted to systematically assess the performance of different DL models in cataract detection and classification from sensitivity, specificity, and the area under the ROC curve (AUC), thereby revealing the methodological and reporting quality and contributing to the clinical translation of DL.

## Methods

### Registration and Study Design

This study adhered to the PRISMA-DTA (Preferred Reporting Items for Systematic Reviews and Meta-analyses of Diagnostic Test Accuracy Studies) [18], and the study protocol was registered on PROSPERO (International Prospective Register of Systematic Reviews; CRD420251030230). We acknowledge that the registration was completed on April 10, 2025, following the initial literature search conducted on April 1, 2025; thus, this is a retrospective registration.

### Search Strategy, Eligibility Criteria, and Data Extraction

Based on the predefined criteria, 2 investigators (RXL and HYL) independently searched Web of Science, IEEE Xplore, Embase, PubMed, and Cochrane Library up to April 1, 2025, for studies published from 2019 to 2025.

The retrieved studies were first imported into EndNote to eliminate duplicate publications. Then the title and abstract were read, and the full text of clearly or potentially eligible studies was examined. No restrictions were imposed on the geographical location or study setting. The following study types were excluded, including letters, non–peer-reviewed reports, narrative reviews, animal studies, and conference abstracts. The search strategy is shown in Multimedia Appendix 1, with specific search phrases, Boolean operations, and field restrictions. The following studies were excluded during full-text screening: studies failing to report key values such as true positives (TP), false positives (FP), false negatives (FN), and true negatives (TN), making it impossible to make a contingency table; studies reporting only composite metrics like sensitivity, specificity, or AUC without providing the underlying data; and studies that reported data ambiguously or in a manner that prevented accurate construction of a 2×2 contingency table. The following is a table of inclusion and exclusion criteria (Table 1). Discrepancies were resolved through discussion with a third investigator (SL or LHL) if needed.

**Table 1.** Eligibility criteria table, which outlines the following aspects: study design, language, data extraction, disease type, and intervention.

Variable	Inclusion criteria	Exclusion criteria
Study design	Randomized controlled trials, prospective observational study, retrospective diagnostic accuracy study, cross-sectional diagnostic study	Letters, non–peer-reviewed reports, narrative reviews, animal studies, and conference abstracts.
Language	Full-text in English	Non-English publications
Data extraction	Studies can report key values such as true positive (TP), false positive (FP), false negative (FN), and true negative (TN), and can create a contingency table. Studies report on comprehensive indicators such as sensitivity, specificity, or AUC, and provide basic data; Studies that report clear data and can accurately construct 2×2 contingency tables.	Studies failing to report key values such as true positives (TP), false positives (FP), false negatives (FN), and true negatives (TN), making it impossible to make a contingency table; Studies reporting only composite metrics like sensitivity, specificity, or AUC without providing the underlying data; Studies that reported data ambiguously or in a manner that prevented accurate construction of a 2×2 contingency table.
Disease type	Cataracts	Noncataract
Intervention	Cataract diagnosis (detection and/or classification)	Noncataract diagnosis

Only studies on the performance of image-based DL algorithms in cataract detection and classification were included following the eligibility criteria (Table 2).

Two investigators (RXL and HYL) independently extracted the following data using standardized data extraction tables: basic characteristics (country, publication year, study site, and type), dataset characteristics (nature, number of images, and presence or absence of external validation), and performance metrics (sensitivity and specificity). Discrepancies were settled by discussion with a third investigator. Contingency tables were used to directly extract the data on binary diagnostic accuracy, including TP, FP, TN, and FN. During data extraction, double-checking was performed, and the original authors were contacted to obtain supplementary data. These data were then used to calculate pooled sensitivity, specificity, and other metrics. If a study provided multiple contingency tables for the same or different DL algorithms, they were assumed to be independent of each other.

**Table 2.** Outlines of the study design and basic demographic characteristics, including authorship details, participant information (number of participants and mean or median age), study design type, inclusion criteria, and exclusion criteria (n=number of participants).

Studies	Participants			N	Median age (range)	Study design
	Inclusion criteria	Exclusion criteria	Labels			
Lin et al (2019) [19]	Patients aged less than 14 years, with or without eye symptoms, and with no history of eye surgery. All participants were required to undergo slit-lamp photography, and sedatives such as chloral hydrate when necessary.	Patients who already had a definitive diagnosis of cataract, other ocular abnormalities, or ocular trauma.	Noncataract or cataract	350	6.58 (6.13-7.03)	Multicenter randomized controlled trial (RCT)
Deepak and Bhat (2024) [20]	NR^a^	NR	Cataract or glaucoma or normal.	NR	NR	Cross-sectional diagnostic study
Zhao et al (2024) [21]	NR	NR	Noncataract or mild cataract or moderate cataract or severe cataract.	NR	NR	Cross-sectional diagnostic study
Zia et al (2023) [22]	NR	NR	Cataract or glaucoma or diabetic retinopathy or neutral.	NR	NR	Retrospective diagnostic accuracy study
Zhang et al (2023) [23]	NR	NR	Normal or low-grade or high-grade.	543	NR	Prospective diagnostic accuracy study
Zeboulon et al (2022) [24]	Patients of either one of the following clinical categories: clear lens or cataract. Patients with clear lens had no history of refractive surgery and had a best corrected visual acuity (BCVA) of at least 20/20. Patients with cataract had significant visual discomfort and were scheduled for surgery. All types of cataracts were included, and 4 experienced cataract and refractive surgeons of the department performed the patient inclusions (authors PZ, CP, WG, and DG).	Patients with any corneal disease.	Normal or cataract or background.	157	NR	Retrospective diagnostic accuracy study
Zhang et al (2024) [25]	NR	NR	NC^b^ severity level: normal or mild or severe.	530	NR	Retrospective diagnostic accuracy study
Zhang et al (2022) [26]	NR	NR	NC severity level: normal or mild or severe.	543	NR	Retrospective diagnostic accuracy study
Xie et al (2023) [27]	Patients with cataract whose best corrected distance visual acuity (BCDVA) was good (>0.6) within 1 month after cataract surgery, and patients without cataract without refractive media opacities. The fundus images were captured without mydriasis before surgery.	Traumatic cataracts, congenital cataracts and lens dislocation, corneal diseases, asteroid hyalosis, vitreous hemorrhage, and severe retinal and optic nerve diseases. Poor quality and unreadable images were also excluded: images out of focus; images underexposed; images overexposed; incomplete images with more than 1/3 peripheral halo.	Noncataract or mild cataracts/ or visually impaired cataracts.	5245	NR	Retrospective diagnostic accuracy study
Wu et al (2022) [28]	NR	Patients with congenital cataract, intraocular lens, aphakic eye, severe eye trauma, or corneal opacity.	Cataract or noncataract with normal-quality images or noncataract with poor-quality images.	30,668	NR	Retrospective diagnostic accuracy study
Vasan et al (2023) [29]	New patients of both the paid and free service facilities who were aged 40 years and older with the BCVA less than 20/40 in either eye. Participants were recruited immediately after the vision examination before further investigation and ophthalmologist examination.	Patients with trauma or vulnerabilities, patients who were unwilling to participate in the study or with dilated pupil.	Negative or positive or “can’t say” or “not asked.”	1407	NR	Prospective diagnostic study
Hassan et al (2024) [30]	NR	NR	Normal or cataract or glaucoma or diabetic or uveitis.	NR	NR	Retrospective diagnostic accuracy study
Ueno et al (2024) [31]	NR	NR	Normal or cataract or infectious keratitis or immunological keratitis or corneal scar or corneal deposits or bullous keratopathy or ocular surface tumor or primary angle-closure glaucoma.	NR	NR	Retrospective diagnostic accuracy study
Singh et al (2024) [32]	NR	NR	Cataract or glaucoma or diabetic retinopathy.	NR	NR	Retrospective diagnostic accuracy study
Shafiq et al (2024) [33]	NR	NR	Glaucoma or cataracts or diabetic retinopathy or myopia or macular degeneration.	NR	NR	Retrospective diagnostic accuracy study
Santone et al (2024) [34]	NR	NR	Normal or cataract.	4785	NR	Retrospective diagnostic accuracy study
Jawad et al (2024) [35]	NR	NR	Normal or glaucoma or cataract or myopia or others.	NR	NR	Retrospective diagnostic accuracy study
Janti et al (2024) [36]	Patients who were aged 45 years and older, and participants of both genders, including males and females.	Patients found to be critically ill after the examination, and those who were not willing to participate in the study.	Cataract positive (mature or immature) or cataract negative (normal and intraocular lens).	495	61.2 (NR-NR）	Prospective, observational diagnostic accuracy study
Emir and Colak (2024) [37]	NR	NR	Healthy or diabetic retinopathy or glaucoma or cataract or age-related macular degeneration or hypertension/myopia or others.	NR	NR	Retrospective diagnostic accuracy study
Ogundokun et al (2024) [38]	NR	NR	AMD^c^ or cataract or diabetes or glaucoma or hypertension myopia or normal.	NR	NR	Retrospective diagnostic accuracy study
Nguyen and Lin (2024) [39]	NR	NR	Cataract or normal.	NR	NR	Retrospective diagnostic accuracy study
Mai et al (2024) [40]	NR	NR	Control (no cataract present), without posterior polar cataract (PPC; cataract present without PPC), with PPC (cataract present with PPC).	103	NR	Retrospective diagnostic accuracy study
Raveenthini et al (2024) [41]	NR	NR	AMD or cataract or diabetic retinopathy or glaucoma or normal.	NR	NR	Retrospective diagnostic accuracy study
Rafay et al (2023) [42]	NR	NR	Cataract or diabetic retinopathy or glaucoma or normal.	NR	NR	Cross-sectional diagnostic study
Abbas et al (2023) [43]	NR	NR	Glaucoma or diabetic retinopathy or cataract or normal.	300	NR	Cross-sectional diagnostic study
Uyar et al (2024) [44]	NR	NR	Cataract or DR or glaucoma or normal.	NR	NR	Retrospective diagnostic accuracy study
Serwaa et al (2024) [45]	NR	NR	Glaucoma-positive or glaucoma-negative or cataracts-positive or cataract-negative.	NR	NR	Retrospective diagnostic accuracy study
Zhang et al (2022) [46]	NR	NR	NC^b^ severity level: normal, mild, or severe.	543	61.30 (42.65-79.95)	Retrospective diagnostic accuracy study
Glaret Subin and Muthukannan (2022) [47]	NR	NR	AMD, diabetic retinopathy, cataract, or glaucoma.	5000	NR	Retrospective diagnostic accuracy study
Xiao et al (2024) [48]	NR	NR	Cortical cataract (CC) severity level: normal, mild, or severe.	469	NR	Retrospective diagnostic accuracy study
Wang et al (2024) [49]	NR	NR	2 coarse-grained types (noncataract No C and posterior subcapsular cataract [PSC]), 7 fine-grained types (NC II, NC III, ≥ NC IV, CC I, CC II, CC III, and CC IV).	NR	NR	Retrospective diagnostic accuracy study
Kumari and Saxena (2024) [50]	NR	NR	Diseased class or normal class.	NR	NR	Retrospective diagnostic accuracy study
Devaraj et al (2024) [51]	Immature cataract, mature cataract, no cataract, and prior cataract operation with intraocular lens (IOL) inserted.	NR	Cataract or noncataract.	7726	50 (NR-NR）	Prospective observational study
Al-Saadi et al (2024) [52]	NR	NR	Normal, early, moderate, or severe.	NR	NR	Retrospective diagnostic accuracy study
Elsawy et al (2023) [53]	Phakic eyes.	Pseudophakic and aphakic eyes.	NC, cortical cataract (cortical lens opacity), or PSC.	2573	69.84 (62.12-77.56)	Retrospective diagnostic accuracy study
Akram and Debnath, 2020 [54]	The symptoms of selected eye diseases include several visual abnormalities in the eye region, particularly blurred, clouded, or yellowing lens, gray or white spots on the cornea, red or bloodshot eyes, yellow or greenish-yellow coatings on eyes, foamy white spots in sclera, swollen eyes, eyelid deformity such as the length of the lower eyelid being turned out from the eye, or reddish bumps on the edge of an inner eyelid depending on specific diseases, and symptoms are different for each disease.	NR	Bitot’s spot of vitamin A deficiency, cataracts, conjunctivitis, corneal ulcer, ectropion, healthy, or periorbital cellulitis or trachoma.	NR	NR	Cross-sectional diagnostic study
Jiang et al (2021) [55]	NR	NR	Opacity area: Limited or Extensive	NR	1.58 (0.7-2.46)	Retrospective diagnostic accuracy study
Yadav and Yadav (2023) [56]	NR	NR	Severity: no, mild, moderate, or severe.	NR	NR	Retrospective diagnostic accuracy study
Yadav and Yadav (2023) [57]	NR	NR	Severity: no, mild, moderate, or severe.	NR	NR	Retrospective diagnostic accuracy study
Subin and Kannan (2022) [58]	NR	NR	Cataract, diabetic retinopathy, glaucoma, or normal.	NR	NR	Cross-sectional diagnostic study
Pratap and Kokil (2019) [59]	NR	NR	Normal, mild, moderate, or severe.	NR	NR	Cross-sectional diagnostic study
Luo et al (2021) [60]	NR	NR	Normal, glaucoma, cataract, or AMD.	NR	NR	Retrospective diagnostic accuracy study
Imran et al (2020) [61]	NR	NR	Normal, mild, moderate, or severe.	NR	NR	Retrospective diagnostic accuracy study
Imran et al (2021) [62]	NR	NR	Normal, mild, moderate, or severe.	NR	NR	Retrospective diagnostic accuracy study
Acar et al (2021) [63]	NR	NR	Noncataract or cataract.	NR	NR	Retrospective diagnostic accuracy study
Olaniyan et al (2024) [64]	NR	NR	Normal or cataract.	NR	NR	Retrospective diagnostic accuracy study
Ganokratanaa et al (2023) [65]	NR	NR	Normal or cataract.	NR	NR	Retrospective diagnostic accuracy study
Gan et al (2023) [66]	NR	NR	Four stages of cataract: incipient stage, intumescent stage, mature stage, or hypermature stage.	NR	NR	Cross-sectional diagnostic study
Tham et al (2022) [67]	NR	Visual impairment caused by other pathologies, incomplete or missing data on cataract grading, or BCVA.	Normal or visually significant cataract.	13,482	NR	Retrospective diagnostic accuracy study
Siddique (2022) [68]	NR	NR	Cataract, chalazion, normal, or squint.	NR	NR	Retrospective diagnostic accuracy study
Sirajudeen et al (2022) [69]	NR	NR	Cataract or normal.	200	NR	Cross-sectional diagnostic study
Junayed et al (2021) [70]	NR	NR	Cataract or noncataract.	NR	NR	Cross-sectional diagnostic study
Hu et al (2020) [71]	NR	NR	Pronounced cataract (2) or early cataract (1) or normal (0).	NR	NR	Retrospective diagnostic accuracy study
Hu et al (2021) [72]	NR	NR	Cataract or normal.	38	58 (NR-NR)	Retrospective diagnostic accuracy study
Lai et al (2022) [73]	NR	NR	Cataract or normal.	NR	NR	Retrospective diagnostic accuracy study
Askarian et al (2021) [74]	NR	NR	Cataract or healthy.	NR	NR	Retrospective diagnostic accuracy study
Son et al, 2022 [75]	Patients had available anterior segment photograph data.	Patients with pathologic features of the cornea, anterior chamber, lens, or iris that interfere with the detection of lens images (eg, corneal opacity or edema, uveitis, and iris defects including aniridia, coloboma, and iridocorneal endothelial syndrome) and a medical history of previous ophthalmic surgery (eg, keratoplasty, implantable Collamer lens, and cataract surgery); patients with retinal and vitreal diseases involving visual pathways that could interfere with visual acuity and final management plan.	Cortical opacity; nuclear color; nuclear opalescence; PSC: normal or mild or moderate or severe.	NR	NR	Cross-sectional diagnostic study
Saju and Rajesh, 2022 [76]	NR	NR	Five types of cataracts: cortical or hyper mature or mature or nuclear or posterior.	NR	NR	Cross-sectional diagnostic study
Chellaswamy et al (2022) [77]	NR	NR	Cataract or diabetic retinopathy or glaucoma or normal or AMD.	NR	NR	Retrospective diagnostic accuracy study
Lu et al (2022) [78]	NR	Eyes with corneal opacity or other corneal disease that might significantly interfere with lens observation and blurred region of interest due to poor fixation or eyes with small pupils that prevented manual cataract evaluation.	Nuclear cataract or cortical cataract or posterior subcapsular cataract.	NR	NR	Retrospective diagnostic accuracy study
Al-Naji et al (2024) [79]	NR	NR	Normal or cataract or foreign body or glaucoma or subconjunctival hemorrhage or viral conjunctivitis.	645	NR	Prospective observational study
Elloumi (2022) [80]	NR	NR	Healthy or mild or moderate or severe cataract.	NR	NR	Retrospective diagnostic accuracy study
Zannah et al (2024) [81]	NR	NR	Cataract or diabetic retinopathy or glaucoma or normal.	NR	NR	Retrospective diagnostic accuracy study

a NR: not reported.

b NC: nuclear cataract.

c AMD: age-related macular degeneration.

### Primary and Secondary Outcomes

Primary and secondary outcomes were defined to assess the performance of DL in cataract detection and classification. The primary outcomes included sensitivity, specificity, and positive and negative likelihood ratios.

The secondary outcomes were used to assess the accuracy of DL versus machine learning (ML) algorithms in cataract diagnosis through subgroup analyses and compare DL algorithms with human experts in studies using identical datasets. The datasets in each study were also investigated for the reference standard used to determine whether transfer learning was applied, the methods for model testing and validation, and the sources and characteristics of the datasets (Table 3).

**Table 3.** Summary of indicators, algorithms, and data sources (n=number of images).

Studies	Indicator definition		Algorithm			Data source			
	Device	Exclusion of poor-quality cases	Algorithm architecture	ML^a^ or DL^b^	Transfer learning applied	Source of data	Number of cases for training or test or internal or external validation	Data range	Open access data
Lin et al (2019) [19]	Slit-lamp photography	Yes	CC-Cruiser	DL	No	ZOC, located in Guangzhou in southern China. The other 4 eye clinics are affiliated with Shenzhen Eye Hospital, the Central Hospital of Wuhan, the Second Affiliated Hospital of Fujian Medical University, and Kaifeng Eye Hospital	NR^c^/350/NR/NR	August 9, 2017-May 25, 2018	NR
Deepak and Bhat (2024) [20]	Retinal fundus camera	No	Darknet-53	DL	Yes	Ocular Disease Intelligent Recognition (ODIR)	4000/1000/NR/NR	NR	ODIR
Zhao et al (2024) [21]	Slit-lamp photography	No	NCME-Net	DL	No	Shenzhen Eye Hospital and the Eye Hospital of Nanjing Medical University	553/100/139/NR	NR	NR
Zia et al (2023) [22]	Retinal fundus camera	No	Improved SqueezeNet model	DL	No	ODIR-IMAGE, Kaggle dataset	1500/400/NR/NR	NR	ODIR-IMAGE, Kaggle dataset
Zhang et al (2023) [23]	CASIA2 AS-OCT ophthalmology device (TOMEY Inc, Japan)	Yes	Ensemble Logistic Regression (EMLR) framework	ML	No	AS-OCT-NC2 dataset	7831/3611/NR/NR	NR	NR
Zeboulon et al (2022) [24]	Swept Source Optical Coherence Tomography (SS-OCT)	No	U-Net model	DL	No	Anterion (Heidelberg)	Development set/validation set: 504/1326	NR	NR
Zhang et al (2024) [25]	CASIA2 AS-OCT ophthalmology device (TOMEY Inc)	No	RCRNets	DL	No	AS-OCT-NC2 dataset	9394/3390/3100/NR	NR	NR
Zhang et al (2022) [26]	CASIA2 AS-OCT ophthalmology device (TOMEY Inc)	Yes	RIR-Net-2‐34	DL	No	The local hospital	7831/3611/NR/NR	NR	UCSD dataset, Heidelberg OCT dataset
Xie et al (2023) [27]	Retinal fundus camera	Yes	DenseNet121^d^	DL	No	Zhejiang Eye Hospital at Wenzhou (ZEHWZ)	4901/1048/1048/1398	September 2020-March 2021	NR
Wu et al (2022) [28]	Retinal fundus camera	Yes	Anti-interference model (convolutional neural network [CNN])	DL	No	The Chinese PLA (People’s Liberation Army) General Hospital	14400/17765/1800/NR	September 2018-May 2021	NR
Vasan et al (2023) [29]	Smartphone camera	Yes	E-Paarvai App (CNN）	DL	No	A large eye care hospital in South India	1400/NR/2619/NR	January 2022-April 2022	NR
Hassan et al (2024) [30]	Retinal fundus camera	No	OcularNET	DL	Yes	The Kaggle machine-learning platform	4000/2200/NR/NR	NR	The Kaggle machine-learning platform.
Ueno et al (2024) [31]	Smartphone camera	Yes	YOLO V.5	DL	No	23 tertiary eye centers in Japan	5270/836/NR/NR	2019‐2020	NR
Singh et al (2024) [32]	Retinal fundus camera	No	A novel ensembled deep learning CNN model	DL	Yes	The Kaggle database	4217/NR/NR/NR	NR	The Kaggle database
Shafiq et al, (2024) [33]	OCT, retinal fundus camera	No	The DualEye-FeatureNet model	DL	No	Structured analysis of the retina (STARE), DRIVE, high-resolution fundus (HRF)	NR/483/NR/NR	NR	STARE, DRIVE, HRF
Santone et al (2024) [34]	Retinal fundus camera	No	The STANDARD_CNN model	DL	No	the ODIR 5K dataset	6987/957/1627/NR	NR	the ODIR 5K dataset
Jawad et al (2024) [35]	Retinal fundus camera	Yes	Swin Transformer models (Swin-T)	DL	No	ODIR, the Retina dataset, available on Kaggle	7000/3400/NR/300	NR	ODIR, the Retina dataset, available on Kaggle
Janti et al (2024) [36]	Smartphone camera	Yes	Smartphone-based cataract screening application	DL	No	The AIIMS (All India Institute of Medical Sciences) Bibinagar, Hyderabad, Telangana, India	NR/990/NR/NR	April 2024-July 2024	NR
Emir and Colak (2024) [37]	Retinal fundus camera	Yes	The residual neural network (ResNet) 50	DL	No	The ODIR dataset	3198/930/471/NR	NR	The ODIR dataset
Ogundokun et al (2024) [38]	Retinal fundus camera	No	MobileNet^e^V2-SVM (support vector machine)	DL	No	Ocular dataset from the Kaggle repository	16290/2012/1811/NR	NR	The Kaggle database
Nguyen and Lin (2024) [39]	Retinal fundus camera	No	Hybrid CNN Approach	DL	Yes	The Kaggle database	888/278/222/NR	NR	The Kaggle database
Mai et al (2024) [40]	A Zeiss OPMI Lumera T surgical microscope	No	ConvNeXt-Tiny model	DL	No	Department of Ophthalmology, Far Eastern Memorial Hospital, New Taipei, Taiwan.	NR/NR/103/NR	January 1, 2018-December 31, 2021	NR
M et al (2024) [41]	Retinal fundus camera	No	XGB classifier model	ML	No	HRF, DR HAGIS, DIARET DB0, DRISHTI, KAGGLE, E-OPTHA, RIM ONE, ORIGA, ACRIMA, DRIONS-DB, STARE, ARIA, IDRID, ICHALLENGE AMD, ODIR, RFMID, KAGGLE CATARACT, HARVARD V1, DERBI DATA, ICHALLENGE, GLAUCOMA	10447/2612/NR/NR	NR	HRF, DR HAGIS, DIARET DB0, DRISHTI, KAGGLE, E-OPTHA, RIM ONE, ORIGA, ACRIMA, DRIONS-DB, STARE, ARIA, IDRID, ICHALLENGE AMD, ODIR, RFMID, KAGGLE CATARACT, HARVARD V1, DERBI DATA, ICHALLENGE, GLAUCOMA
Rafay et al (2023) [42]	Retinal fundus camera	No	EfficientNet^f^ B3	DL	Yes	The Kaggle database	2949/1268/NR/NR	NR	The Kaggle database
Abbas et al (2023) [43]	Retinal fundus camera	No	Deep-ocular mode	DL	Yes	The retinal fundus multidisease image dataset (RFMiD) and ODIR	1222/521/NR/NR	NR	RFMiD and ODIR
Uyar et al (2024) [44]	Retinal fundus camera	No	ABC-based weighted ensemble model	DL	Yes	The Eye Disease Dataset (EDD), from the Kaggle	3372/426/419/NR	NR	EDD, from the Kaggle
Serwaa et al (2024) [45]	Retinal fundus camera	No	LBPSCN: Local Binary Pattern Scaled Capsule Network	DL	No	The Kaggle database	NR	NR	The Kaggle database
Zhang et al (2022) [46]	CASIA2 AS-OCT ophthalmology device (TOMEY Inc)	Yes	Clinical-awareness attention network (CCA-Net)	DL	No	A local health physical center	9619/3141/3441/NR	NR	The ACRIMA dataset, the UCSD dataset
Glaret Subin and Muthukannan (2022) [47]	Fundus camera	No	FPOA-CNN	DL	No	Various medical centers in China collected by the Shanggong Medical Technology Co., Ltd.	NR	NR	ODIR database
Xiao et al (2024) [48]	CASIA2 AS-OCT ophthalmology device (TOMEY Inc)	No	ResNet34-MSSA	DL	No	CASIA2 AS-OCT dataset	3969/1271/1305/NR	NR	The LAG dataset, the SD-OCT dataset
Wang et al, 2024 [49]	Slit-lamp photography	No	MGCNet	DL	No	The Cataract Center of Beijing Tongren Hospital (BTH; Beijing, China)	2912/970/970/NR	NR	The APTOS2019 dataset, The HAM10000 dataset
Kumari and Saxena (2024) [50]	Retinal fundus camera	No	RINet	DL	No	Multiple repositories	NR	NR	NR
Devaraj et al (2024) [51]	Smartphone camera	No	EfficientNet-v2 Model	DL	No	The ophthalmology departments at King George’s Medical University (KGMU) and Balrampur Hospital, Lucknow	1708/753/275/NR	October 29, 2022-September 23, 2023	NR
Al-Saadi et al (2024) [52]	Retinal fundus camera	No	An Automated Wavelet Scattering Network	DL	No	The ODIR database	357/155/NR/NR	NR	The ODIR database
Elsawy et al (2023) [53]	Color fundus photography (CFP)	No	Deep Opacity Net	DL	Yes	The AREDS2 dataset, the Singapore Epidemiology of Eye Diseases study (SEED)	12227/3514/1773/17088	2006‐2008	NR
Akram and Debnath (2020) [54]	Digital camera	No	A deep convolution neural network (DCNN) model	ML	No	International Center for Eye Health, clinical images for symptoms on faces from the University of Rochester, UCSD School of Medicine and VA Medical Center, the Primary Care Dermatology Society, and other different resources	1402/350/NR/NR	NR	NR
Jiang et al (2021) [55]	Slit-lamp photography	No	CCNN-Ensemble	DL	Yes	Zhongshan Ophthalmic Center of Sun Yat-sen University	470 (Training and validation)/132/79	June 2015-February 2020	NR
Yadav and Yadav (2023) [56]	Retinal fundus camera	Yes	CNN with 2D DFT	DL	No	HRF, STARE, MESSIDOR, DRIVE, DRIONS_DB, and IDRiD datasets, as well as images obtained from the internet	NR	NR	HRF, STARE, MESSIDOR, DRIVE, DRIONS_DB, and IDRiD datasets
Yadav and Yadav (2023) [57]	Retinal fundus camera	No	CNN with ensemble of SVM, NB, RF	DL	No	HRF, STARE, MESSIDOR, DRIVE, DRIONS_DB, and IDRiD databases, as well as other images collected from the internet	NR	NR	HRF, STARE, MESSIDOR, DRIVE, DRIONS_DB, and IDRiD databases
Subin and Kannan (2022) [58]	Retinal fundus camera	No	AMSO-RNN (recurrent neural network) Model	DL	No	ODIR database	2240/960/NR/NR	NR	ODIR database
Pratap and Kokil (2019) [59]	Retinal fundus camera	Yes	Pre-trained CNN	TL	Yes	HRF image database, STARE, standard diabetic retinopathy database (DIARETDB0), e-ophtha: a color fundus image database, MESSIDOR database, DRIVE database, fundus image registration (FIRE) dataset, digital retinal images for optic nerve segmentation database (DRIONS-DB), IDRiD, available datasets from Dr Hossein Rabbani, and other internet resources	400/400/NR/NR	NR	HRF image database, STARE, DIARETDB0, e-ophtha: a color fundus image database, MESSIDOR database, DRIVE database, FIRE dataset, DRIONS-DB, IDRiD, available datasets from Dr Hossein Rabbani, and other internet resources
Luo et al (2021) [60]	Retinal fundus camera	No	FCL-EfficientNet-B3	DL	No	Shanggong Medical Technology Co, Ltd. OIA-ODIR dataset	1000/274/NR/NR	NR	OIA-ODIR dataset
Imran et al (2020) [61]	Retinal fundus camera	No	The combination of DL models (AlexNet, ResNet, and VGG^g^Net) and SVM	DL	Yes	The Tongren Hospital, China	6424/1607/NR/NR	NR	NR
Imran et al (2021) [62]	High-resolution fundus camera Canon-EOS-40D with additional settings such as 72 DPI resolution, no-flash, manual exposure, and auto-white balance	No	A novel hybrid method, namely CRNN, based on CNN and RNN	DL	Yes	The Tongren Hospital, China	6424/1606/NR/NR	NR	NR
Acar et al (2021) [63]	Retinal fundus camera	No	VGGNet	DL	Yes	The Kaggle Ocular Disease Recognition database	3891/1216/973/NR	NR	The Kaggle Ocular Disease Recognition database
Olaniyan et al (2024) [64]	Slit-lamp photography	No	Hybrid Siamese-VGG16 model	DL	No	Kaggle’s public repository	NR/121/NR/NR	NR	Kaggle’s public repository
Ganokratanaa et al (2023) [65]	Slit-lamp photography	No	LeNet-CNN	DL	No	NR	5600/1400/NR/NR	NR	NR
Gan et al (2023) [66]	Slit-lamp photography	No	Automatic segmentation DTL platform	DL	No	Department of Ophthalmology, Jiangxi Provincial People’s Hospital	517/130/nr/nr	NR	NR
Tham et al (2022) [67]	Retinal fundus camera	Yes	The ResNet-50	DL	No	Singapore Eye Research Institute	8045/1692/NR/16005	NR	NR
Siddique (2022) [68]	Photos from phones and internet	No	MobileNet	DL	No	4 hospitals from Bangladesh	1762/439/NR/NR	NR	NR
Sirajudeen et al (2022) [69]	Retinal fundus camera	No	Novel Kernel-based CNN	DL	No	The Kaggle database	320/80/NR/NR	NR	The Kaggle database
Junayed et al (2021) [70]	Retinal fundus camera	No	CataractNet	DL	No	Multiple databases	904/226/NR/NR	NR	Multiple databases
Hu et al (2020) [71]	Smartphone with slit-lamp	No	UDFA (Faster-RCNN)^h^	DL	No	Marked Slit Lamp Picture Project (MSLPP)	11272/819/4831/NR	NR	MSLPP
Hu et al (2021) [72]	The iSpector-mini mobile phone slit lamp developed by Shenyang EyeROBO Intelligent Technology Co, Ltd.	No	ACCV	DL	No	A cooperation hospital	1064/304/152/NR	NR	NR
Lai et al (2022) [73]	Digital camera	No	CNNDCI	DL	No	GitHub.com	7735/193/89/NR	NR	GitHub.com
Askarian et al (2021) [74]	Smartphone camera	No	SVM	ML	No	NR	63/30/7/NR	NR	NR
Son et al (2022) [75]	Slit-lamp photography	No	An ensemble of 3 AI algorithms: ResNet18, WideResNet50-2, and ResNext50	DL	Yes	Local outpatient clinic	2706/792/446/NR	January 2017-December 2020	NR
Saju and Rajesh (2022) [76]	Slit-lamp photography	No	Dense CNN+BE_ResNet101 classification model	DL	No	The DRIMDB dataset, various hospitals	NR/264/NR/NR	NR	The DRIMDB dataset
Chellaswamy et al (2022) [77]	Retinal fundus camera	No	WODCNN method	DL	No	KAGGLE, MESSIDOR, ORIGA, DRIVE, STARE datasets	1661/414/NR/NR	NR	KAGGLE, MESSIDOR, ORIGA, DRIVE, STARE datasets
Lu et al (2022) [78]	Slit-lamp photography	Yes	Faster R-CNN and ResNet	DL	No	An internal dataset from the EENT Hospital of Fudan University and an external dataset from the Pujiang Eye Study	964/214/156/NR	An internal dataset of slit lamp photographs of the anterior segment of cataract-affected eyes taken between 2018 and 2020. Another external dataset of slit lamp photographs taken between March 2018 and August 2019.	NR
Al-Naji et al (2024) [79]	Retinal fundus camera	No	InceptionResNetV2	DL	No	The Balad Ruz General Hospital and Ibn Al-Haitham Teaching Eye Hospital	453/194/NR/NR	January 2, 2023-July 7, 2023	NR
Elloumi (2022) [80]	Retinal fundus camera	No	Ensemble Learning (InceptionV3, MobileNet-V2, and NasNet-Mobile)	DL	Yes	the Kaggle platform	354/118/118/NR	NR	The “Cataract Dataset,” “Ocular Disease Recognition (ODiR)”
Zannah et al (2024) [81]	Retinal fundus camera	No	BayeSVM500 model	ML	Yes	Cataract dataset, Glaucoma dataset, High-Resolution Fundus (HRF) Image Database, Kaggle, IEEE-Dataport, and Pattern Recognition Lab	4144/1037/NR/NR	NR	The Kaggle database, High-Resolution Fundus (HRF) Image Database

a ML: machine learning.

b DL: deep learning.

c NR: not reported.

d DenseNet: dense convolutional network.

e MobileNet: efficient convolutional neural networks for mobile vision apps.

f EfficientNet: rethinking model scaling for convolutional neural networks.

g VGG: visual geometry group.

h Faster-R CNN: fast region–based convolutional network.

### Quality Assessment

Three investigators independently assessed the risk of bias (RoB) and applicability concerns in the clinical context in the included studies by using the Quality Assessment of Diagnostic Accuracy Studies-2 (QUADAS-2) [82]. Cross-validation was implemented to enhance interrater agreement, and the methodology for consistency assessment was explicitly reported. Deeks’ funnel plots were drawn to evaluate the publication bias if more than 10 studies were included. Statistical significance was set at *P*<.05. Deeks’ funnel plot asymmetry test was performed using the Deeks command within the *MIDAS* package in STATA 18.0 (StataCorp LLC).

### Statistical Analysis

STATA 18.0 and RevMan 5.4 (Review Manager; The Cochrane Collaboration) were used for data analyses. The summary receiver operating characteristic (SROC) curve plotting, assessment of heterogeneity, and analysis of publication bias were performed using STATA 18.0 to enhance transparency and reproducibility. The heterogeneity was assessed by the Cochran Q test and *I*² statistic. The threshold of the *I*² statistic for quantifying heterogeneity proposed by Higgins et al [83] was adopted: *I*²≤25%: low, *I*²≈50%: moderate, *I*²≥75%: substantial. This strategy was specifically designed to identify whether inconsistencies in study results stemmed from random factors or reflected substantive discrepancies. When significant heterogeneity (*P*<.05 or *I*²>50%) was identified [84], a bivariate mixed effects model was adopted. The bivariate random-effects model was fitted using the *MIDAS* package in STATA 18.0. This method demonstrated particular efficacy in synthesizing pooled estimates of sensitivity, specificity, and AUC across studies. Its advantage resides in the capability to systematically address metric variability while preserving the intrinsic correlation.

To evaluate the accuracy of the DL algorithm, a hierarchical SROC curve was fitted. We calculated corresponding 95% CIs for sensitivity, specificity, and AUC using the Delta method, which linearized the nonlinear relation of the log-transformed sensitivity and specificity by a first-order Taylor expansion. Then the variance-covariance matrix of the parameter estimates was propagated, whereas prediction intervals (PIs) incorporated between-study heterogeneity by modeling the covariance structure of sensitivity and specificity [84,85]. Sensitivity analyses were conducted on all DL algorithms to be evaluated, rather than only the one with the highest accuracy. A random-effects model was used to explain potential between-study variability.

### Ethical Considerations

This study required no informed consent or ethical approval. Data previously collected from human subjects in ethical/institutional review board–approved studies were used. All studies included adhered to the Declaration of Helsinki.

## Results

### Search Results

**Figure 1.**
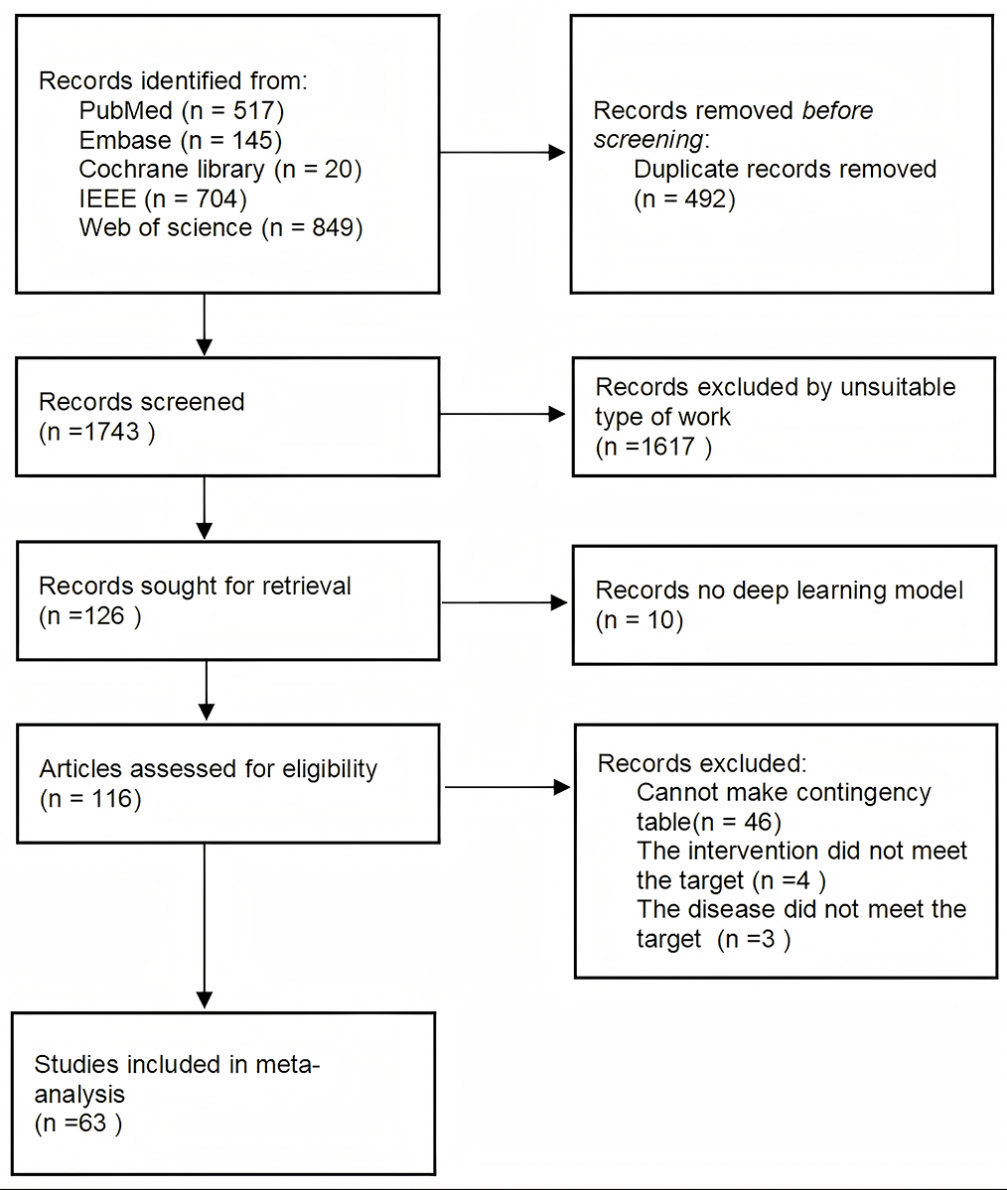
PRISMA (Preferred Reporting Items for Systematic Reviews and Meta-Analyses) flowchart of study selection. The PRISMA flow of search methodology and literature selection process is displayed.

Initially, 2235 studies were retrieved, of which 492 duplicates were excluded. Following study screening, 1680 studies were excluded from quantitative synthesis (meta-analysis), including 1617 studies involving animal research, nondisease studies, surgical technique investigations, reviews, and conference reports; 10 studies not using deep learning algorithms; 46 studies lacking sufficient data for constructing 2×2 contingency tables or reporting data in formats incompatible with pooling (eg, AUC only); 4 studies not focusing on cataract diagnostic models; and 3 studies that did not address cataract disease (Figure 1; Checklist 1).

### Study Characteristics

As shown in Tables 2-4, all the included studies were published in 2019‐2024, involving 171,416 images. Retrospective data were used in 46 studies, prospective data in only 5 studies, and cross-sectional data in 12 studies. The data came from open-access sources in 31 studies. The sample size was prespecified in one study, and low-quality images were excluded in 15 studies. External validation was conducted in 12 studies, while the remainder performed internal validation. Six studies compared DL with traditional ML models using the same dataset, while another 6 compared DL models with human experts. Forty-four studies focused on cataract detection, 17 on cataract classification, and 2 on both detection and classification. Cataract detection was categorized as binary detection (presence vs absence of cataracts, n=21) and multidisease detection (n=25). Moreover, cataracts were classified into mild (n=18), moderate (n=11), and severe (n=19) types. Additionally, 49 studies did not describe cataract classification; among the other studies, clinical subtypes included posterior subcapsular cataract (PSC; n=4), pediatric cataract (n=2), posterior polar cataract (PPC; n=1), nuclear cataract (NC; n=10), and cortical cataract (CC; n=6).

**Table 4.** Author information, reference standards, types of internal validation, and whether external validation is applied.

Studies	Reference standard	Type of internal validation	External validation
Lin et al (2019) [19]	Expert consensus, consistent label	Hold-out cross-validation	No
Deepak and Bhat (2024) [20]	Clinical diagnosis by ophthalmologists; validated by trained readers	5-fold cross-validation	No
Zhao et al (2024) [21]	AREDS No 4 guidelines	5-fold cross-validation	No
Zia et al (2023) [22]	Clinical diagnosis by ophthalmologists; validated by trained readers	5-fold cross-validation	No
Zhang et al (2023) [23]	The LOCS III^a^ system	Hold-out cross-validation	No
Zeboulon et al (2022) [24]	The cataract fraction (CF)	5-fold cross-validation	Yes
Zhang et al (2024) [25]	The LOCS III system	Hold-out cross-validation	Yes
Zhang et al (2022) [26]	The LOCS III system	Hold-out cross-validation	Yes
Xie et al (2023) [27]	Cataract specialists	Hold-out cross-validation	Yes
Wu et al (2022) [28]	Cataract specialists	Hold-out cross-validation	Yes
Vasan et al (2023) [29]	Slit lamp diagnosis with dilated eyes by an ophthalmologist	Hold-out cross-validation	No
Hassan et al (2024) [30]	Clinical diagnosis by ophthalmologists; validated by trained readers	Hold-out cross-validation	No
Ueno et al (2024) [31]	Corneal specialists	Hold-out cross-validation	No
Singh et al (2024) [32]	Clinical diagnosis by ophthalmologists; validated by trained readers	Hold-out cross-validation	No
Shafiq et al (2024) [33]	Clinical diagnosis by ophthalmologists	NR^b^	No
Santone et al (2024) [34]	Clinical diagnosis by ophthalmologists (based on electronic medical records and verified by trained readers)	Hold-out cross-validation	No
Jawad et al (2024) [35]	Clinical diagnosis by ophthalmologists; validated by trained readers	Hold-out cross-validation	Yes
Janti et al (2024) [36]	The LOCS III system	NR	No
Emir and Colak (2024) [37]	Clinical diagnosis by ophthalmologists	Hold-out cross-validation	No
Ogundokun et al (2024) [38]	Clinical diagnosis by ophthalmologists; validated by trained readers	Hold-out cross-validation	No
Nguyen and Lin (2024) [39]	Clinical diagnosis by ophthalmologists; validated by trained readers	5-fold cross-validation	No
Mai et al (2024) [40]	Visual inspection of the surgical view of the cataract in the surgery video	5-fold cross-validation	No
Raveenthini et al (2024) [41]	Clinical diagnosis by ophthalmologists	ten-fold cross-validation	No
Rafay et al (2023) [42]	Clinical diagnosis by ophthalmologists; validated by trained readers	Hold-out cross-validation	No
Abbas et al (2023) [43]	Clinical diagnosis by ophthalmologists	Hold-out cross-validation	No
Uyar et al (2024) [44]	Clinical diagnosis by ophthalmologists; validated by trained readers	10-fold cross-validation	No
Serwaa et al (2024) [45]	Clinical diagnosis by ophthalmologists; validated by trained readers	NR	No
Zhang et al (2022) [46]	Experienced ophthalmologists	NR	Yes
Glaret Subin and Muthukannan (2022) [47]	Clinical diagnosis by ophthalmologists; validated by trained readers	10-fold cross-validation	No
Xiao et al (2024) [48]	Clinical grading by ophthalmologists	NR	No
Wang et al (2024) [49]	Clinical diagnosis by ophthalmologists	Hold-out cross-validation	No
Kumari and Saxena (2024) [50]	Clinical diagnosis by ophthalmologists	5-fold cross-validation	No
Devaraj et al (2024) [51]	Clinical diagnosis by ophthalmologists	Hold-out cross-validation	No
Al-Saadi et al (2024) [52]	Clinical diagnosis by ophthalmologists; validated by trained readers	Hold-out cross-validation	No
Elsawy et al (2023) [53]	The Wisconsin Cataract Grading System, the AREDS2 NS severity scale.	Hold-out cross-validation	Yes
Akram and Debnath (2020) [54]	Clinical grading by ophthalmologists	10-fold cross-validation	No
Jiang et al (2021) [55]	Three senior ophthalmologists	5-fold cross-validation	Yes
Yadav and Yadav (2023) [56]	A professional ophthalmologist	NR	No
Yadav and Yadav (2023) [57]	Clinical diagnosis by ophthalmologists	NR	No
Subin and Kannan (2022) [58]	Clinical diagnosis by ophthalmologists; validated by trained readers	Hold-out cross-validation	No
Pratap and Kokil (2019) [59]	Clinical diagnosis by ophthalmologists; validated by trained readers	Hold-out cross-validation	No
Luo et al (2021) [60]	Trained ophthalmologists	5-fold cross-validation	No
Imran et al (2020) [61]	Two retinal experts	5-fold cross-validation	No
Imran et al (2021) [62]	Clinical diagnosis by ophthalmologists	5-fold cross-validation	No
Acar et al (2021) [63]	Clinical diagnosis by ophthalmologists	Monte-Carlo cross-validation	No
Olaniyan et al (2024) [64]	Clinical diagnosis by ophthalmologists; validated by trained readers	Hold-out cross-validation	No
Ganokratanaa et al (2023) [65]	NR	5-fold cross-validation	No
Gan et al (2023) [66]	Experienced ophthalmologists	5-fold cross-validation	No
Tham et al (2022) [67]	Wisconsin cataract grading system or AREDS system	5-fold cross-validation	Yes
Siddique (2022) [68]	Clinical grading by ophthalmologists	5-fold cross-validation	No
Sirajudeen et al (2022) [69]	Clinical diagnosis by ophthalmologists; validated by trained readers	5-fold cross-validation	No
Junayed et al (2021) [70]	Composite reference standard (Expert clinical diagnosis or grading from original source datasets)	5-fold cross-validation	No
Hu et al (2020) [71]	Ophthalmologists with more than 5 years of clinical experience	Hold-out cross-validation	No
Hu et al (2021) [72]	The LOCS III system	Hold-out cross-validation	No
Lai et al (2022) [73]	The LOCS III system	5-fold cross-validation	Yes
Askarian et al (2021) [74]	NR	10-fold cross-validation	No
Son et al (2022) [75]	The LOCS III system	Hold-out cross-validation	No
Saju and Rajesh (2022) [76]	Clinical diagnosis by ophthalmologists	Hold-out cross-validation	No
Chellaswamy et al (2022) [77]	Clinical diagnosis by ophthalmologists	Hold-out cross-validation	No
Lu et al (2022) [78]	The LOCS III system	Hold-out cross-validation	Yes
Al-Naji et al (2024) [79]	Clinical grading by ophthalmologists	Hold-out cross-validation	No
Elloumi (2022) [80]	Clinical diagnosis by ophthalmologists; validated by trained readers	5-fold cross-validation	No
Zannah et al (2024) [81]	Clinical diagnosis by ophthalmologists; validated by trained readers	5-fold cross-validation	No

a LOCS III: Lens Opacities Classification System III.

b NR: not reported.

### Pooled Performance of DL Algorithms

**Figure 2.**
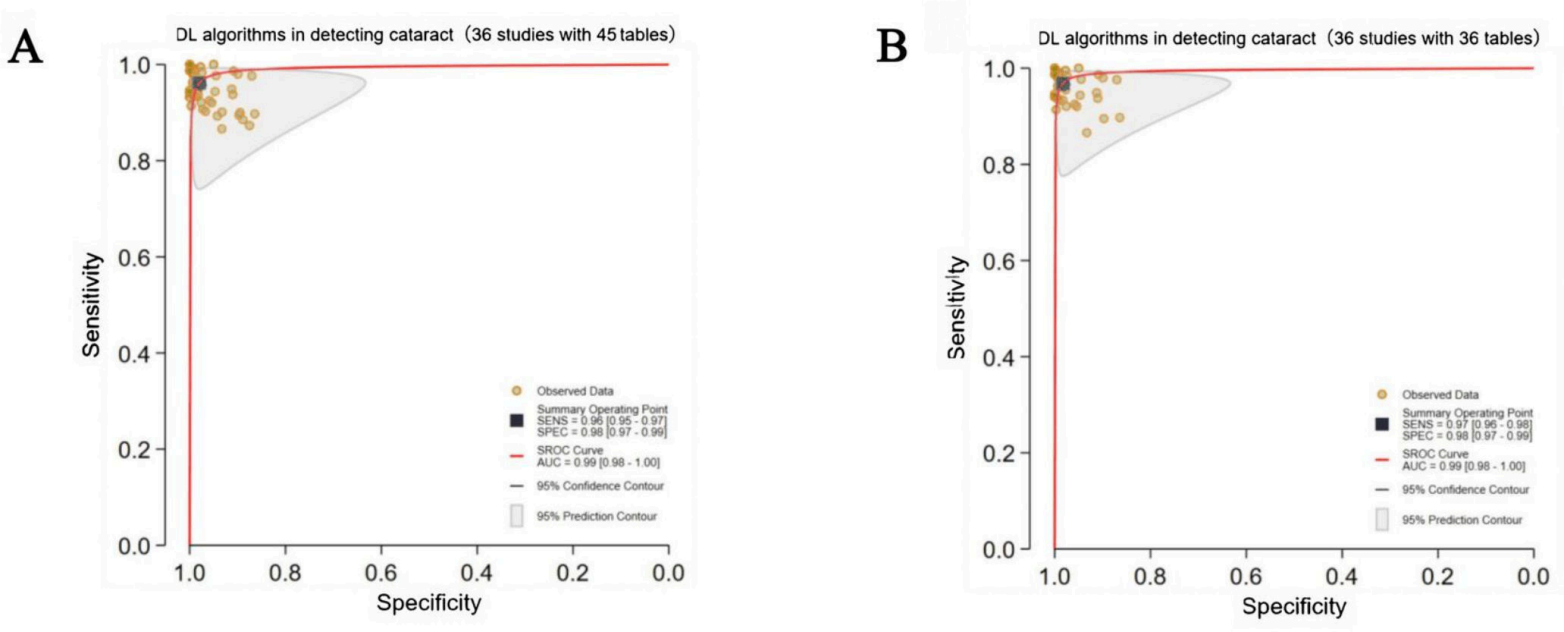
Pooled overall performance of deep learning (DL) algorithms for cataract detection. (A) Receiver operating characteristic (ROC) curves of all studies included in the meta-analysis (36 studies with 45 tables). (B) ROC curves of studies reporting the highest accuracy (36 studies with 36 tables). The cataract detection is divided into binary detection and multidisease detection. AUC: area under the ROC curve; SROC: summary receiver operating characteristic.

Finally, 63 studies [19-81] with sufficient data (97 contingency tables) were included for the assessment of DL performance in cataract detection and classification [86]. Hierarchical SROC curves for cataract detection (45 contingency tables) and classification (52 contingency tables) are provided in Figures2A  3A, respectively. The classification task involved multiclassification (eg, mild, moderate, and severe cataracts), and separate SROC curves were generated for each category. For cataract detection, the pooled sensitivity and specificity of DL were 96% (0.95‐0.97) and 98% (0.97‐0.99), respectively, with an AUC of 0.99 (0.98‐1.00). For cataract classification, DL had pooled sensitivity and specificity of 94% (0.93‐0.96) and 97% (0.96‐0.98), respectively, with an AUC of 0.99 (0.98‐1.00). Great heterogeneity and inconsistency were observed across cataract severity (mild: *I*²=99%, moderate: *I*²=96%, severe: *I*²=99%; *P*<.001), suggesting substantial variability in diagnostic or methodological methods across studies. 25 contingency tables for mild cataracts were used in 18 studies, 16 contingency tables for moderate cataracts in 11 studies, and 28 contingency tables for severe cataracts in 19 studies, which may introduce classification imbalance, potentially influencing the performance assessment.

**Figure 3.**
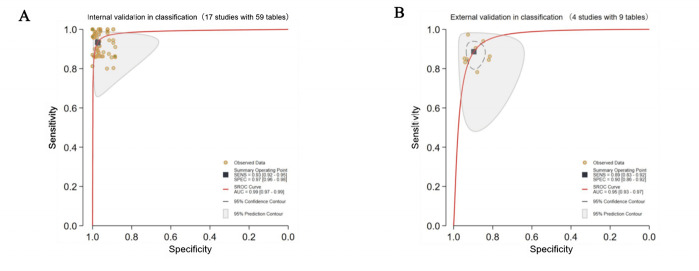
Pooled overall performance of deep learning (DL) algorithms for cataract classification. (A) Receiver operating characteristic (ROC) curves of all studies included in the meta-analysis (17 studies with 52 tables); (B) ROC curves of studies reporting the highest accuracy (17 studies with 17 tables). The cataract classification is divided by severity (mild, moderate, and severe) and clinical subtypes. AUC: area under the ROC curve; DL: deep learning; SROC: summary receiver operating characteristic.

One or more DL algorithms were reported in most studies, and DL with the highest accuracy was selected, ultimately obtaining 53 contingency tables. For cataract detection, DL had pooled sensitivity and specificity of 97% (96%‐98%) and 98% (97%‐99%), respectively, with an AUC of 0.99 (0.98‐1.00) (Figure 2B). For cataract classification, the pooled sensitivity and specificity were 95% (0.92‐0.97) and 98% (0.96‐0.99), respectively, with an AUC of 0.99 (0.98‐1.00) (Figure 3B). Threshold analyses were conducted using STATA 18.0 to investigate threshold effects. For diagnostic models in primary studies that did not prespecify a threshold, sensitivity and specificity corresponding to the optimal threshold reported in the study were extracted. If a primary study reported results for multiple thresholds, we prioritized extracting and analyzing data corresponding to the threshold associated with the reported primary endpoint or optimal operating point. The spatial distribution of classification thresholds in ROC curves was analyzed; each threshold corresponded to a unique point on the ROC curve, and systematic evaluation of these points could help us quantify performance variability across thresholds and detect threshold-driven instability (defined as significant performance fluctuations within narrow threshold ranges). The SROC curve displayed no “shoulder-arm” distribution (Figures2  3).

However, the model’s performance in independent external datasets (detection: sensitivity 87%, specificity 93%; classification: sensitivity 89%, specificity 90%) was lower than the overall estimates. Notably, the lower performance observed in external validation datasets compromised the generalization capability of the model, potentially attributable to domain shift, warranting caution when applied to new populations or settings.

### Subgroup Analyses

#### Overview

Traditional ML or DL algorithms were reported in the included studies. These studies varied in primary objectives (detection, classification, or both detection and classification). Due to overlapping objectives among studies, the sum of the number of studies on traditional ML and DL algorithms did not match the total number of included studies. According to the Lens Opacities Classification System III (LOCS III) [87] and the methods of Mackenbrock et al [88] and Gali et al [89], we classified cataracts into mild, moderate, and severe types and further categorized cataracts into PSC, pediatric cataract, PPC, NC, and CC.

#### Detection

##### Algorithm Types

**Figure 4.**
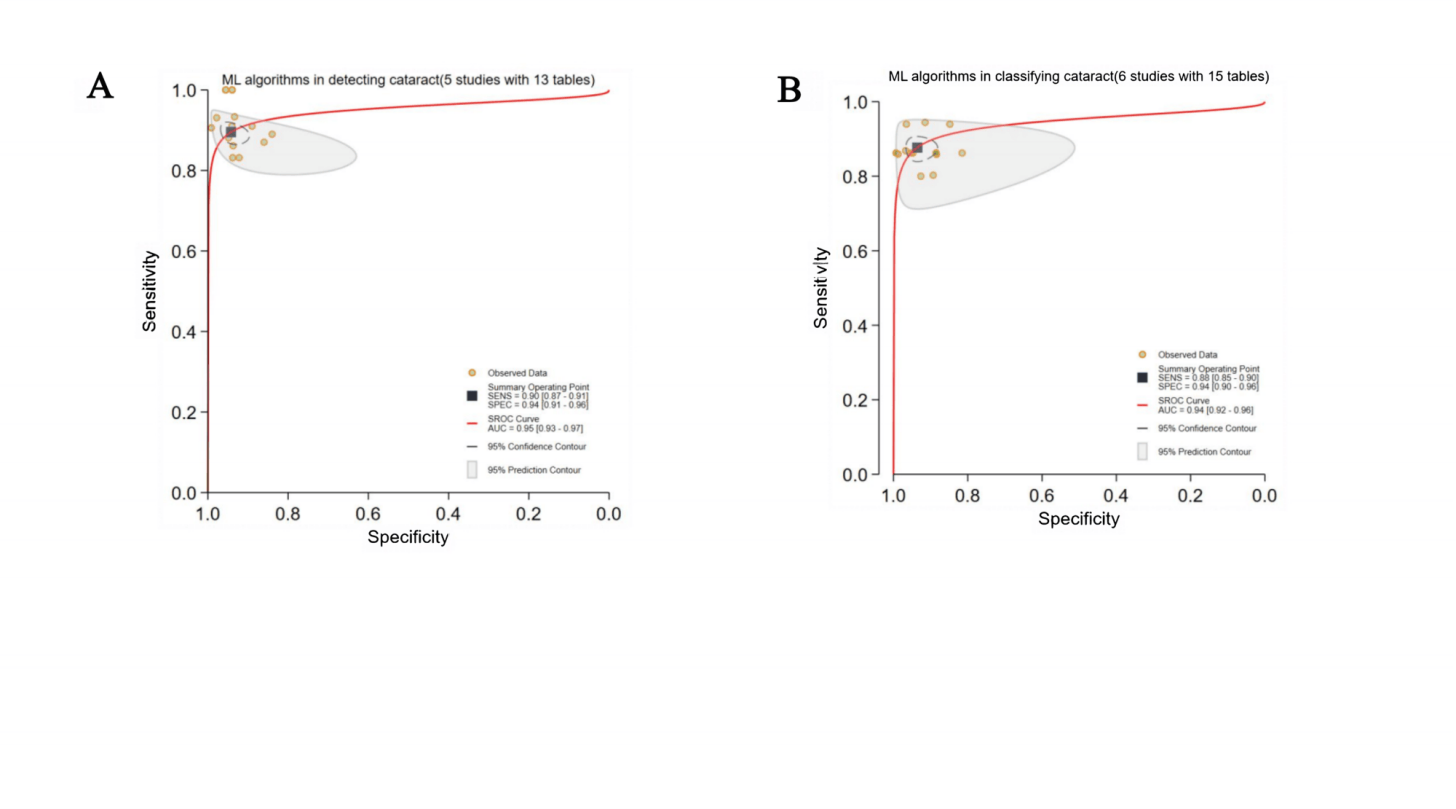
Pooled overall performance of (no-DL) machine learning (ML) algorithms. (A) Receiver operating characteristic (ROC) curves of studies using ML algorithms for cataract detection (5 studies with 13 tables); (B) ROC curves of studies using ML algorithms to classify cataracts (6 studies with 15 tables). AUC: area under the ROC curve; ML: machine learning; SROC: summary receiver operating characteristic.

DL algorithms were described in 36 studies (45 contingency tables). The pooled sensitivity and specificity of DL were 96% (95%‐97%) and 98% (97%‐99%), respectively, with an AUC of 0.99 (0.98‐1.00; Figure 2A). Additionally, traditional ML algorithms were described in 5 studies (13 contingency tables). The pooled sensitivity and specificity of ML were 90% (87%‐91%) and 94% (91%‐96%), respectively, with an AUC of 0.95 (0.93‐0.97; Figure 4A).

##### Disease Types

**Figure 5.**
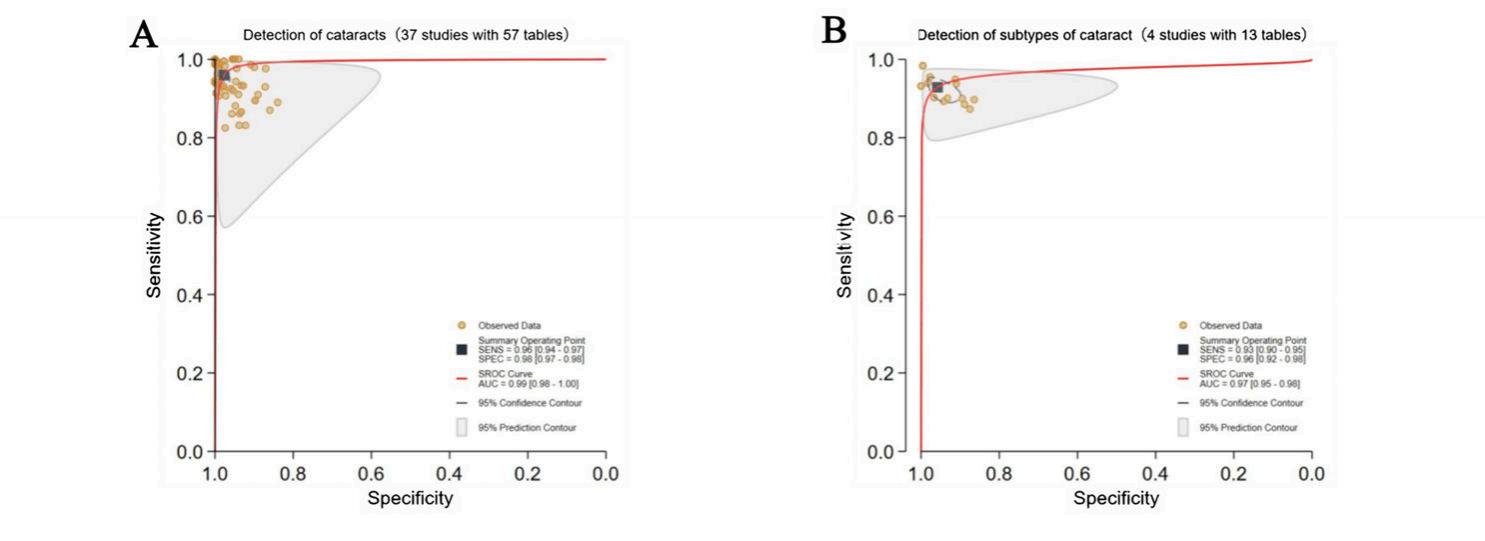
Pooled performance based on disease types in cataract detection. (A) Receiver operating characteristic (ROC) curves of studies detecting unclassified cataracts (37 studies with 57 tables); (B) ROC curves of studies detecting subtypes of cataract (4 studies with 13 tables). AUC: area under the ROC curve; SROC: summary receiver operating characteristic.

Around 37 studies (57 contingency tables) focused on nonspecific or general cataracts; the pooled sensitivity, specificity, and AUC were 96% (94%‐97%), 98% (97%-98%), and 0.99 (0.98‐1.00). The Cochran Q test revealed a statistically significant result (Q=199.023; *P*<.001) and high inconsistency (*I*²=99%; 95% CI 98‐99), indicating significant between-study variability. For cataract detection (CC: one study with one contingency table, pediatric cataract: 2 studies with eight contingency tables, NC: 2 studies with 3 contingency tables, PSC: one study with one contingency table, PPC: one study with one contingency table), the pooled sensitivity and specificity were 93% (90%‐95%) and 96% (92%‐98%), respectively, and the AUC was 0.97 (0.95‐0.98) in 4 studies (13 contingency tables). Due to the extremely limited sample sizes across clinical subtypes, with most subtypes reported by only one study, the meta-analysis results were less robust. Consequently, only a pooled heterogeneity assessment could be conducted. The Cochran Q test revealed a statistically significant result (Q=9.167; *P*=.005) and high inconsistency (*I*²=78%; 95% CI 53‐100), indicating significant between-study variability (Figures 5A and 5B).

##### Validation Types

**Figure 6.**
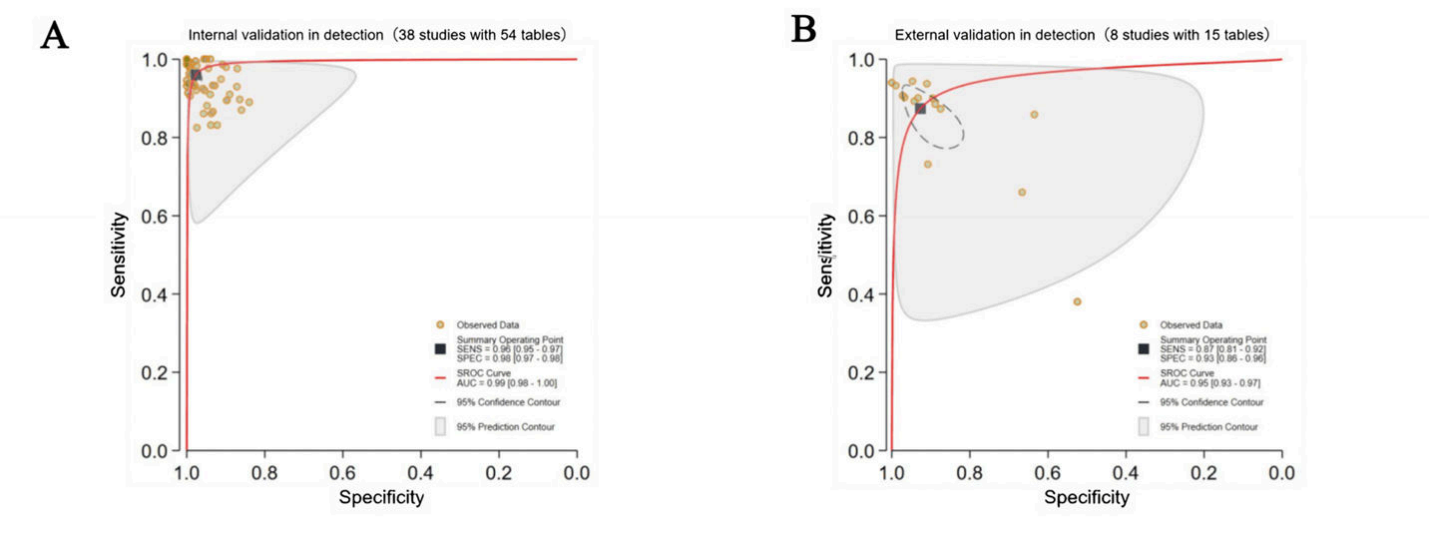
Pooled performance based on validation types in cataract detection. (A) Receiver operating characteristic (ROC) curves of studies with internal validations (38 studies with 54 tables); (B) ROC curves of studies with external validations (8 studies with 15 tables). AUC: area under the ROC curve; SROC: summary receiver operating characteristic.

Great heterogeneity and inconsistency were observed in the validation methods for cataract detection (*I*²=99% for both internal and external validation; *P*<.001), suggesting substantial variability in diagnostic or methodological methods across studies. The Deeks’ funnel plots revealed no great publication bias in the internal validation (*P*=.68) or the external validation (*P*=.23). The internal validation was conducted in 38 studies (54 contingency tables), which showed a pooled sensitivity of 96% (95%‐97%) and a pooled specificity of 98% (97%-98%), with an AUC of 0.99 (0.98‐1.00). The external validation was performed in only 8 studies (15 contingency tables), which showed a pooled sensitivity of 87% (81%‐92%) and a pooled specificity of 93% (86%‐96%), respectively, with an AUC of 0.95 (0.93‐0.97; Figures 6A and 6B).

### Classification

#### Algorithm Types

DL algorithms were described in 17 studies (52 contingency tables); the pooled sensitivity and specificity of DL were 94% (93%‐96%) and 97% (96%‐98%), respectively, with an AUC of 0.99 (0.98‐1.00; Figure 3A). Additionally, traditional ML algorithms were described in 6 studies (15 contingency tables); the pooled sensitivity and specificity of ML were 88% (85%‐90%) and 94% (90%‐96%), respectively, with an AUC of 0.94 (0.92‐0.96; Figure 4B).

#### Disease Types

**Figure 7.**
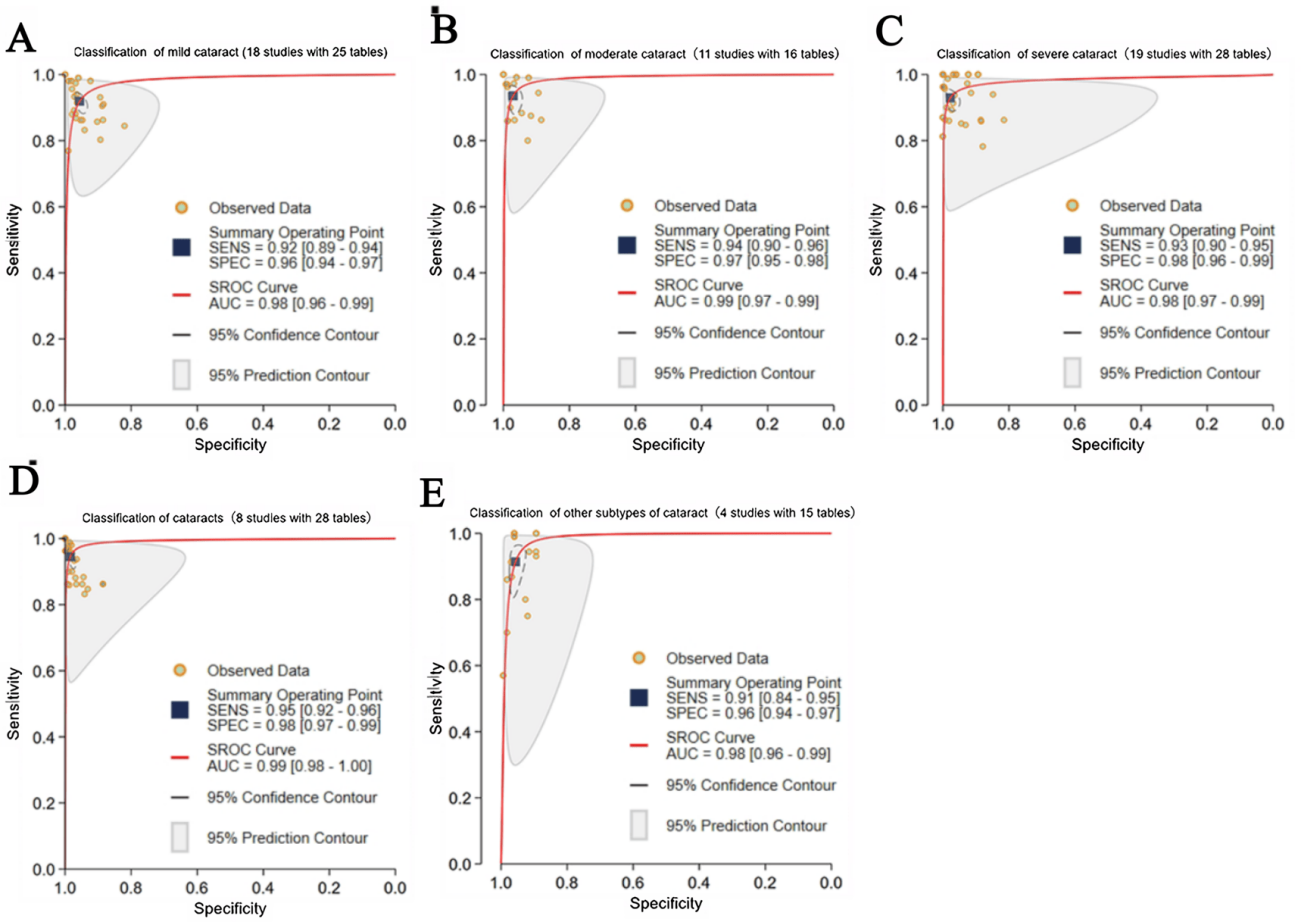
Pooled performance based on disease types in cataract classification. (A) receiver operating characteristic (ROC) curves of studies on classification of mild cataract (18 studies with 25 tables); (B) ROC curves of studies on classification of moderate cataract (11 studies with 16 tables); (C) ROC curves of studies on classification of severe cataract (19 studies with 28 tables); (D) ROC curves of studies on classification of cataracts (8 studies with 28 tables); (E) ROC curves of studies on classification of other subtypes of cataract (4 studies with 15 tables). AUC: area under the ROC curve; SROC: summary receiver operating characteristic.

For mild cataracts, 18 studies (25 contingency tables) had a pooled sensitivity of 92% (89‐94%) and a pooled specificity of 96% (94%‐97%), with an AUC of 0.98 (0.96‐0.99). For moderate cataracts, 11 studies (16 contingency tables) found comparable performance: SE=94% (90%‐96%), specificity=97% (95%‐98%), and AUC=0.99 (0.97‐0.99). For severe cataracts, 19 studies (28 contingency tables) had a sensitivity of 93% (90%‐95%) and a specificity of 98% (96%‐99%), with an AUC of 0.98 (0.97‐0.99). Great heterogeneity and inconsistency were observed across cataract severity (mild: *I*²=99%, moderate: *I*²=96%, severe: *I*²=99%; *P*<.001), suggesting substantial variability in diagnostic or methodological methods across studies. Furthermore, eight studies with 28 contingency tables focused on nonspecific or general cataracts, which had a pooled sensitivity of 95% (92%‐96%) and a pooled specificity of 98% (97%‐99%), with an AUC of 0.99 (0.98‐1.00). Great heterogeneity and inconsistency were observed across cataract severity (nonspecific or general cataract: *I*²=96%, NC: 100%; *P*<.001), suggesting substantial variability in diagnostic or methodological methods across studies. In the NC subgroup (9 studies with 27 contingency tables), extremely high heterogeneity was observed (*I*²=100%). Consequently, to maintain statistical consistency, we did not calculate a pooled estimate for this subgroup. Instead, the descriptive analysis showed a sensitivity of 89%‐93% and a specificity of 93%‐97% across these studies. Four studies with 15 contingency tables considered other clinical subtypes (PSC: one study with 3 contingency tables; CC: 4 studies with 12 contingency tables), with pooled sensitivity, specificity, and AUC of 91% (84%‐95%), 96% (94%‐97%), and 0.98 (0.96‐0.99). Due to the extremely limited sample sizes of other clinical subtypes, the meta-analysis results were less robust. Consequently, only a pooled heterogeneity assessment could be conducted. The Cochran Q test revealed a statistically significant result (Q=120.355; *P*<.001) and high inconsistency (*I*²=98%; 95% CI 97‐99), indicating significant between-study variability (Figures 7A-7E).

#### Validation Types

**Figure 8.**
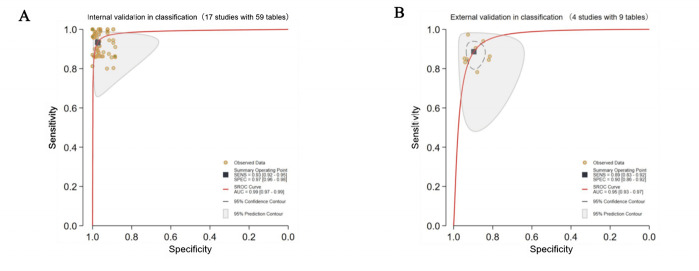
Pooled performance based on validation types in cataract classification. (A) Receiver operating characteristic (ROC) curves of studies with internal validations (17 studies with 59 tables); (B) ROC curves of studies with external validations (4 studies with 9 tables). AUC: area under the ROC curve; SROC: summary receiver operating characteristic.

The internal validation was adopted in 17 studies (59 contingency tables), which showed a pooled sensitivity of 93% (92%-95%) and a pooled specificity of 97% (96%‐98%), with an AUC of 0.99 (0.97‐0.99). The external validation was adopted in only 4 studies (9 contingency tables), which showed a pooled sensitivity of 89% (83%‐92%) and a pooled specificity of 90% (86%‐92%), with an AUC of 0.95 (0.93‐0.97; Figure 8).

### Comparison Between DL and ML Algorithms

#### Detection

**Figure 9.**
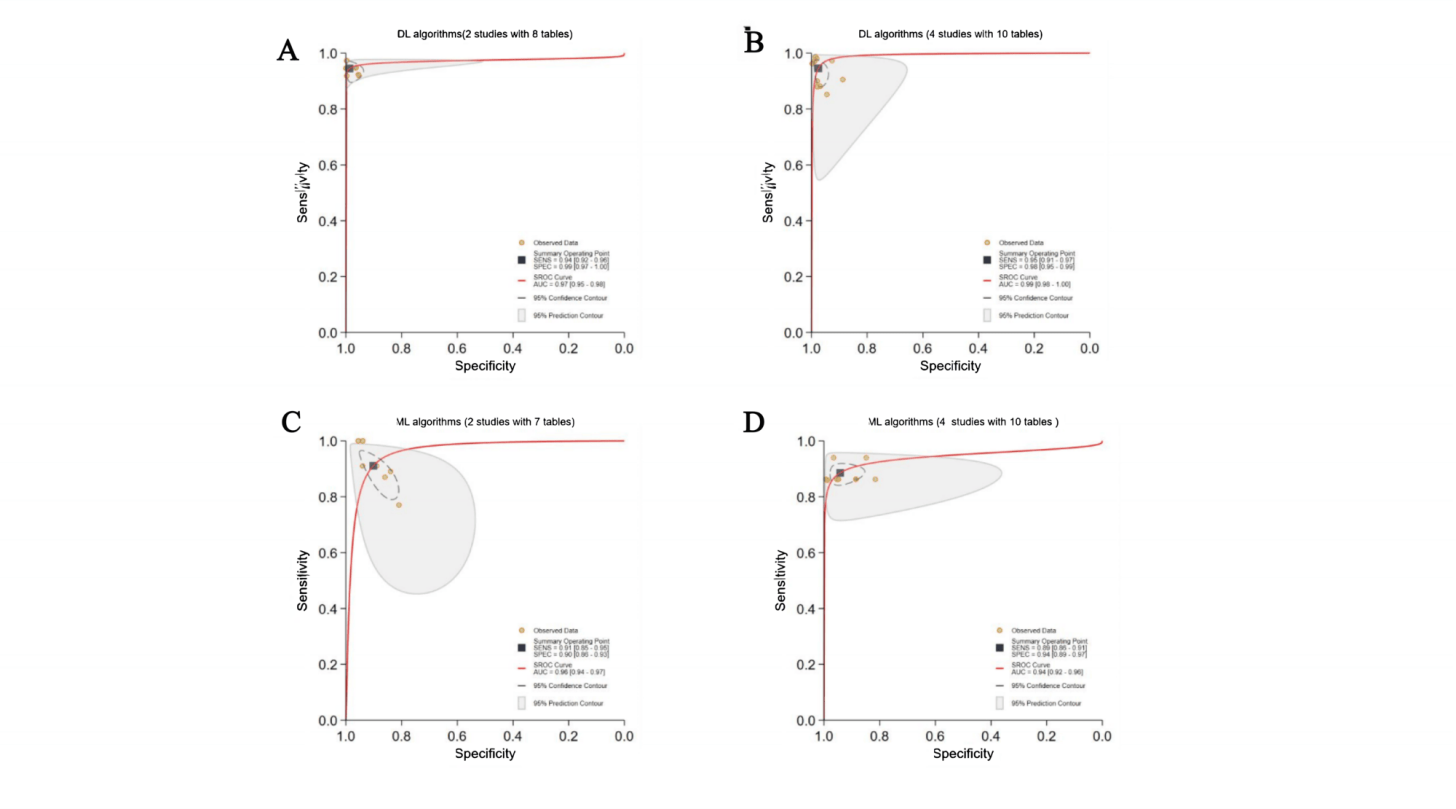
Pooled subgroup performance of deep learning (DL) versus machine learning (ML) algorithms. AUC: area under the ROC curve; SROC: summary receiver operating characteristic.

Two studies compared DL (8 contingency tables) and ML algorithms (7 contingency tables) using the same dataset. The pooled sensitivity was 94% (92%‐96%) for DL and 91% (85%‐95%) for ML algorithms. The pooled specificity was 99% (97%‐100%) for DL and 90% (86%‐93%) for ML algorithms. The AUC was 0.97 (0.95‐0.98) for DL and 0.96 (0.94‐0.97) for ML algorithms. For DL algorithms, the Cochran Q test revealed a statistically significant result (Q=9.675; *P*=.004) and high inconsistency (*I*²=79%; 95% CI 55‐100). For ML algorithms, the Cochran Q test also revealed a statistically significant result (Q=5.853; *P*=0.03) and high inconsistency (*I*²=66%; 95% CI 23‐100). Therefore, both DL and ML exhibited significant heterogeneity. However, due to overlapping CIs and no direct between-group comparisons, the statistical significance of the between-group heterogeneity could not be conclusively established based on these findings. The comparison between DL and ML algorithms for cataract detection was constrained at the time of this writing by minimal validation evidence, with only 2 studies available for direct benchmarking. As a result, it severely limited statistical power, compromised generalization capability, and inflated performance estimates for DL models (Figures 9A and 9B).

#### Classification

Four studies compared DL (10 contingency tables) and ML algorithms (10 contingency tables) using the same dataset. The pooled sensitivity was 95% (91%‐97%) for DL and 89% (86%‐91%) for ML algorithms. The pooled specificity was 98% (95%‐99%) for DL and 94% (89%‐97%) for ML algorithms. The AUC was 0.99 (0.98‐1.00) for DL and 0.94 (0.92‐0.96) for ML algorithms (Figures 9C and 9D).

### DL Algorithms Versus Human Experts

**Figure 10.**
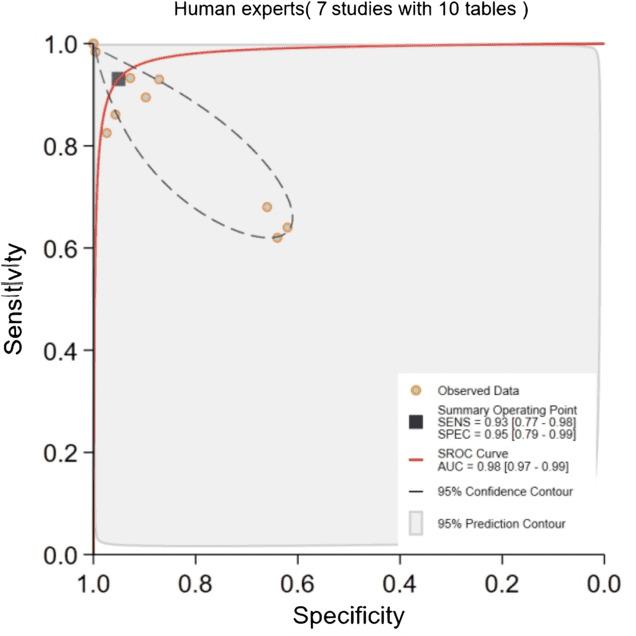
Pooled performance of human experts. AUC: area under the ROC curve; SROC: summary receiver operating characteristic.

Direct comparisons between DL algorithms (7 contingency tables) and human experts (10 contingency tables) were performed across 7 studies using the same datasets. For DL algorithms, quantitative pooling was not conducted due to extreme heterogeneity (Q=933.852; *P*<.001; *I*²=100%). Instead, a descriptive analysis revealed highly variable performance of DL algorithms, with sensitivity estimates ranging from 72% to 93% and specificity ranging from 64% to 99%. In contrast, human experts demonstrated moderate heterogeneity (Q=5.811; *P*=0.03; *I*²=66%), allowing for a pooled analysis. The pooled sensitivity and pooled specificity for human experts were 93% (95% CI 77%‐98%) and 95% (95% CI 79%‐99%), respectively, with an AUC of 0.98 (95% CI 0.97‐0.99; Figure 10). These results highlight that DL algorithms exhibited significantly higher heterogeneity than human experts. The Deeks’ funnel plot indicated no significant publication bias for human experts (*P*=.16), but potential borderline publication bias for DL algorithms (*P*=.05). The extreme heterogeneity (*I*²=100%) observed in the DL algorithms underscores that their efficacy is highly dependent on specific study conditions and architectures, precluding a uniform performance metric.

### Publication Bias and Heterogeneity

**Figure 11.**
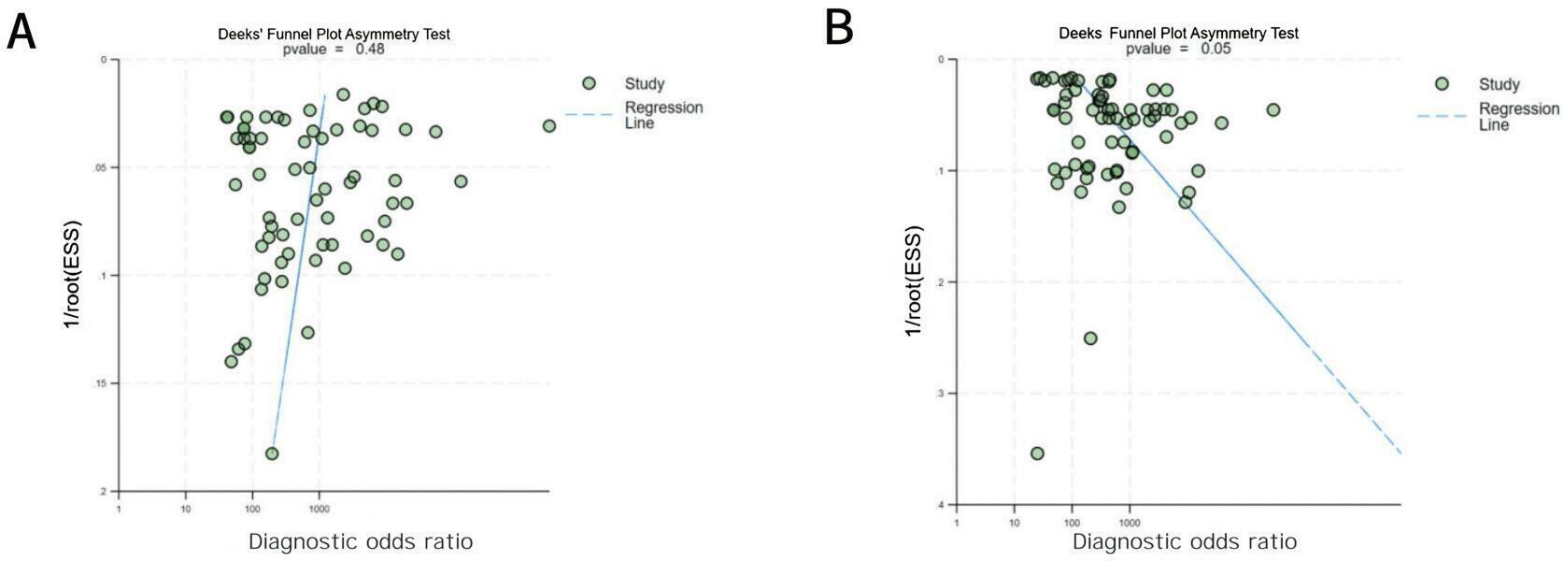
Summary estimate of pooled performance using funnel plots. (A) Deeks’ funnel plot asymmetry test of studies detecting cataracts; (B) Deeks’ funnel plot asymmetry test of studies classifying cataracts.

The Deeks’ funnel plot revealed no significant publication bias for cataract detection (*P*=.48). However, potential borderline publication bias was detected for cataract classification (*P*=.05). However, the wide distribution of studies near the regression line in the plot should be further considered (Figures 11A and 11B).

**Figure 12.**
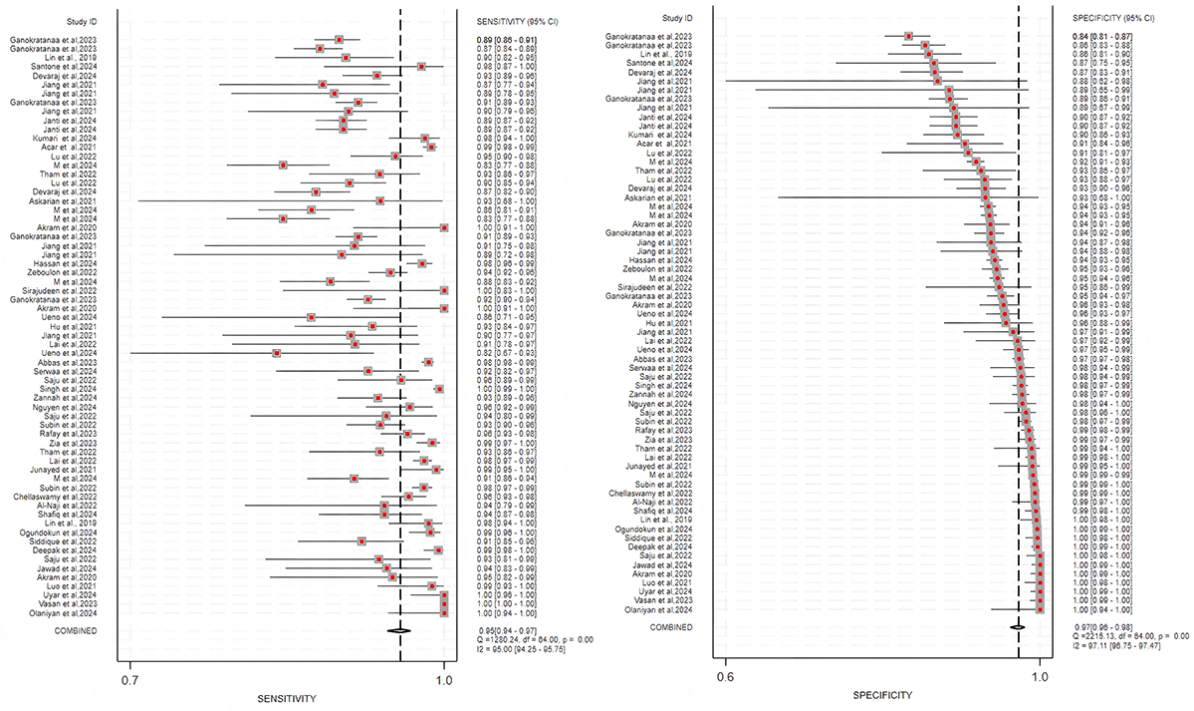
Summary estimate of pooled performance using forest plots. The forest plot of studies for the cataract detection (41 studies).

The included studies showed substantial heterogeneity. The high *I*² values (sensitivity: 95.00% and specificity: 97.11% for cataract detection; sensitivity: 95.94% and specificity: 98.55% for classification) suggested considerable between-study variability (*P*<.001), which was further explored by subgroup analyses (Figures12  13).

**Figure 13.**
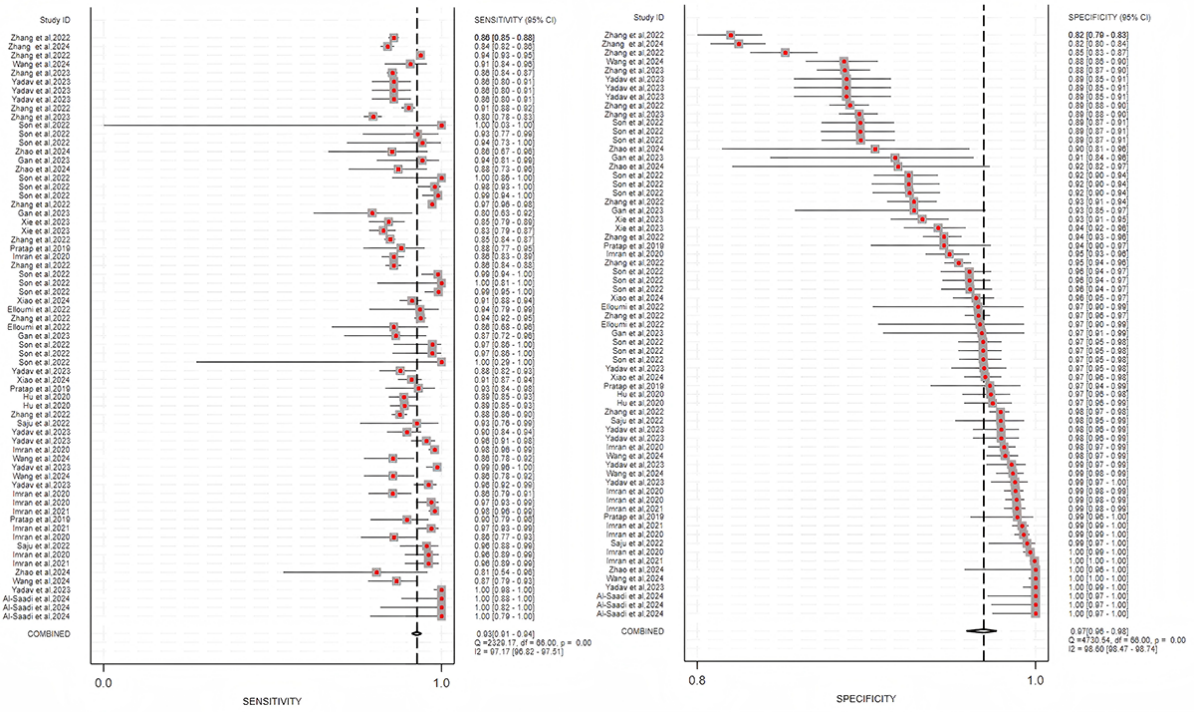
Summary estimate of pooled performance using forest plots. The forest plot of studies for the cataract classification (19 studies).

### RoB

The quality of included studies was assessed using QUADAS-2, and a summary of findings is displayed in Figure S1 in Multimedia Appendix 1 (summary of methodological quality of 63 studies included). A detailed assessment for each item of RoB and applicability concern is also provided in Figure S2 in Multimedia Appendix 1 (diagrams of methodological quality of 63 studies included). High RoB or selection bias was detected in 34 studies due to a lack of randomization or eligibility criteria in the patient selection domain; RoB was high or unclear in 44 studies due to no predefined threshold in the index test domain.

Due to inconsistencies in reference standard (no reporting of whether blinding was implemented and the presence or absence of a predefined threshold), RoB was high or unclear in 2 studies. RoB was high or unclear in 34 studies in the domain of flow and timing due to no mention of whether the same gold standard was used or the presence or absence of an appropriate time gap.

High or unclear applicability in the domain of patient selection was detected in one study and unclear applicability in the domain of index test in 5 studies; 2 studies showed applicability concerns in the domain of reference standard.

## Discussion

This meta-analysis included 63 studies and rigorously assessed the study quality using the QUADAS-2 to summarize the evidence to date on the performance of image-based DL in cataract diagnosis. The results revealed that DL algorithms might offer higher accuracy than traditional ML algorithms and fall within the range of reported accuracy of human experts in the detection of cataracts, demonstrating potential as tools for automated diagnosis. However, given the moderate quality and high heterogeneity of the current evidence base, these DL algorithms are considered primarily as adjuncts to cataract diagnosis.

DL has made tremendous progress in automated image analysis [6]. In clinical practice, a severe imbalance between supply and demand is present in ophthalmologic diagnosis. As AI advances, DL is expected to raise diagnostic efficiency and thus help alleviate health care resource inequality.

Four relevant systematic reviews and meta-analyses were identified. (1) Cheung et al [90] found high sensitivity, specificity, and reproducibility and great heterogeneity of the ML model for cataract diagnosis in children and adults, but the strength of evidence was limited since only 11 studies with 13 contingency tables were included. (2) Liu et al [91] found comparable performance of DL and experts in medical imaging, but only 2 out of 18 ophthalmology studies involved cataracts, necessitating in-depth research on DL in cataract diagnosis. (3) Aggarwal et al [92] included 82 ophthalmology studies, which did not involve cataracts, and verified that DL possesses good sensitivity, specificity, and AUC for the feature identification of other eye diseases, but the algorithmic evaluation criteria remain to be standardized due to high heterogeneity. (4) Islam et al [93] demonstrated that DL can be applied to retinal vessel segmentation, so it can be popularized in LMICs. Therefore, methodological limitations should be critically assessed during the clinical translation of DL to further improve its reliability.

This meta-analysis systematically evaluated the effectiveness of DL versus traditional ML in cataract detection and classification to provide a basis for clinical decision-making. For cataract detection, DL had a pooled sensitivity of 96% (95% CI 95%‐97%) and a pooled specificity of 98% (97%‐99%), with an AUC of 0.99 (0.98‐1.00); traditional ML had a pooled sensitivity of 90% (87%‐91%) and a pooled specificity of 94% (91%‐96%), with an AUC of 0.95 (0.93‐0.97). For cataract classification, the pooled sensitivity and specificity of DL were 94% (93%-96%) and 97% (96%‐98%), with an AUC of 0.99 (0.98‐1.00); the pooled sensitivity and specificity of traditional ML were 88% (85%‐90%) and 94% (90%‐96%), with an AUC of 0.94 (0.92‐0.96). Available evidence suggests that DL models exhibit high sensitivity and specificity in automated cataract diagnosis and that medical image-based DL still demonstrates superior robustness to ML despite the RoB in most studies. However, our conclusions were made partly based on studies of low quality due to a lack of external validation and nonstandardized reporting of performance metrics, which may overestimate algorithmic accuracy. The use of overlapping public repositories introduced potential clustering bias. To ensure robust generalizability, future research must prioritize validation on independent, multicenter, and nonpublic datasets. Furthermore, the lack of a sensitivity analysis excluding high-RoB studies implied that the high overall performance of DL models could be partially influenced by methodologically weaker studies, warranting validation in future high-quality trials. Finally, due to the limited number of eligible studies, we pooled smartphone-based data from varying modalities, including diffuse photography and slit-lamp adapters. While this aligned with our assessment of general mobile health accessibility, it introduced potential optical heterogeneity. Consequently, statistical quantification of performance differences between optical sectioning and diffuse lighting was not feasible, warranting future separate investigations.

We identified the potential of DL for clinical use from the included studies, but the number of studies directly comparing the performance of DL and ML in cataract diagnosis was limited, with only 2 in detection and 4 in classification. Traditional ML (eg, logistic regression, random forest, and support vector machine) is dependent on manual feature engineering and classifiers, whose performance is limited by feature quality and domain knowledge [94]. By contrast, DL (eg, CNN and transformer-based vision models) has higher accuracy in image recognition by automatic feature extraction but requires higher data volume and stronger arithmetic support [95].

The number of included studies was limited, so image segmentation performance was not meta-analyzed. Image segmentation is essentially a pixel-level prediction task, which relies on the ability to characterize discriminative features. However, it is often difficult for traditional ML to effectively capture such features. In contrast, DL demonstrates significantly better performance by automated learning of complex patterns in highly heterogeneous data [6]. The property of DL models is such that they contain millions of parameters that need to be optimized by data-driven training, while traditional ML models display higher stability in small-sample multiclassification [95]. These findings suggest that traditional ML models may have the potential to raise the accuracy in cataract classification, while DL is more advantageous in reducing misdiagnosis rates. A combination of the 2 may facilitate the clinical translation of AI in the future.

Despite the available evidence on the clinical translational potential of DL, only 7 studies compared the efficacy of DL and human experts in cataract detection, and no studies compared their performance in cataract classification. Notably, significant interrater variability was present in expert performance due to heterogeneity in the cumulative clinical experience and health care resource allocation, highlighting the need for more comprehensive comparative studies. These studies generally report positive results of DL, but an optimism bias in algorithm performance may be produced due to insufficient sample sizes and methodological heterogeneity. Therefore, it is urgently needed to adopt a standardized study design and transparent reporting norms for improving the quality of evidence and clinical translational value of DL. DL has been successfully applied to retinal fundus image analysis [14] for automated diagnosis of DR [96] and glaucoma [97]. Considering the technical feasibility of DL in ophthalmic disease screening, it is recommended that DL serve as an adjunct to clinical diagnosis to optimize the treatment process by human-machine collaboration. Additionally, no great publication bias was detected, but the findings need to be interpreted with caution. It is recommended that a clinician’s diagnostic efficiency be used as a core assessment metric and that a real-world validation be incorporated in subsequent studies.

In the field of AI methodology, several standardized guidelines have been recently issued [98,99]. However, no unified consensus has been reached on AI for cataract diagnosis. At the time of this writing, computer-aided diagnostic techniques mainly use a combination of medical image processing and AI, but research focuses mostly on eye diseases of the posterior segment (eg, DR, and age-related macular degeneration) and less on the anterior segment (eg, cataracts). Nowadays, cataract diagnosis relies on slit-lamp microscopy of lens morphology, and cataract classification is based on the LOCS III [87], the Oxford Clinical Cataract Classification and Grading System [100], and the American Cooperative Cataract Research Group method [101]. DL has made a preliminary breakthrough in automated cataract classification, such as the turbidity-density-location assessment system developed by Lin et al [19] based on anterior segment slit lamp images and the DL classification model developed by Zhou et al [102] using retinal fundus images, but a standardized severity scale adapted to DL is urgently needed for its clinical use. Therefore, it is recommended that future studies standardize the criteria for performance assessment of DL algorithms, with a focus on improving the transparency of methodology reporting and repeatability validation process.

Data scarcity and lack of generalization capability are key scientific challenges for DL. In this systematic review, retrospective designs were adopted in most of the included studies, while prospective designs and multicenter clinical trials accounted for less than 5%, and their annotation criteria were not optimized for the need of DL, restricting methodological rigor. We should recognize that most of the studies used double-blind, annotated, and quality-controlled image data for model training, which effectively improved diagnostic accuracy and reduced the RoB while balancing data size and quality. The included studies generally used data enhancement techniques, indicating the lack of high-quality annotated datasets and prospective validation studies.

The acquisition of representative data for clinical validation remains the major bottleneck. The model’s clinical generalization capability is severely restricted due to the long time consumption of pixel-level annotation of fundus images, domain shift resulting from cross-device and cross-race variations, and a lack of fine-grained pathology characterization. In the future, research should focus on multimodal data fusion for modeling (eg, OCT plus fundus images), deployment of edge computing architectures, and embedding of causal inference modules, thereby establishing intelligent diagnosis and treatment systems with both clinical credibility and engineering practicality.

In this study, great methodological heterogeneity was found in the reporting of DL performance metrics, including incomplete reporting of sensitivity or specificity, a lack of 2×2 contingency tables, and general overreliance on aggregated metrics such as AUC-ROC and *F*_1_-score. Of particular note is that high AUC values (>0.90) may mask the risk of misjudgment of key positive events (eg, progressive cataract) in clinical scenarios with a severe imbalance in category distribution. Based on these findings, it is recommended that the confusion matrix serve as a core reporting metric. The above-mentioned problems can be gradually settled by high-quality studies.

Another key obstacle is no consensus on the interpretability of DL decision-making mechanisms. It is difficult to intuitively understand the decision-making logic of DL models due to their complex network structure and nonlinear feature extraction, which, as a “black-box” characteristic, has been deemed a challenge for clinical use. Recently, researchers have gradually revealed the intrinsic mechanism of DL models by gradient-weighted class activation mapping, adversarial testing, and causal inference [95]. For example, Chang et al [103] used adversarial samples to analyze the decision-making basis of DL models in glaucoma detection. Araújo et al [104] located the key region of DR in fundus images by multiple-instance learning. Explainable AI (XAI) is breaking through the limitations of traditional AI by synchronizing decision results with attributional explanations [105]. Abràmoff et al [106] systematically reviewed the interpretability framework for DL models in the medical field, laying a theoretical foundation for their clinical translation. Future research needs to further explore the innovative methods of XAI in medical image analysis to enhance clinical credibility.

This study showed that DL was applied primarily to the screening of eye diseases such as DR for which mature diagnostic guidelines have been established. Notably, differences in health care resource allocation should be considered when popularizing DL. In subsequent studies, the clinical effect and health economic benefits should be assessed across different DL algorithms, and the “black-box” problem of DL algorithms should be solved using interpretability methods to enhance clinical acceptance.

## Supplementary material

10.2196/78869 Multimedia Appendix 1 Methodological quality summary and risk of bias graph of the studies included in the meta-analysis.

10.2196/78869 Checklist 1 PRISMA checklist.

## Abbreviations

AI: artificial intelligence
AUC: area under the receiver operating characteristic curve
CC: cortical cataract
CNN: convolutional neural network
DL: deep learning
DR: diabetic retinopathy
FDA: the US Food and Drug Administration
FN: false negative
FP: false positive
*ICD-11*: *International Classification of Diseases, 11th Revision*
LMIC: low- and middle-income country
LOCS III: lens opacities classification system III
LRS: low-resource settings
ML: machine learning
NC: nuclear cataract
OCT: optical coherence tomography
PI: prediction interval
PPC: posterior polar cataract
PRISMA-DTA: Preferred Reporting Items for Systematic Reviews and Meta-analyses of Diagnostic Test Accuracy
PROSPERO: International Prospective Register of Systematic Reviews
PSC: posterior subcapsular cataract
QUADAS-2: Quality Assessment of Diagnostic Accuracy Studies-2
ResNet: residual network
RevMan: Review Manager
RoB: risk of bias
SROC: summary receiver operating characteristic curve
TN: true negative
TP: true positive
WHO: World Health Organization
XAI: explainable artificial intelligence
