# Supplementary material for: Image-Based Deep Learning for Cataract Diagnosis: Systematic Review and Meta-Analysis
Source: J Med Internet Res. 2026 Apr 29;28:e78869. doi: 10.2196/78869 (PMC13128160; doi:10.2196/78869)
Supplement: Multimedia Appendix 1 [file jmir-v28-e78869-s001.doc]

**
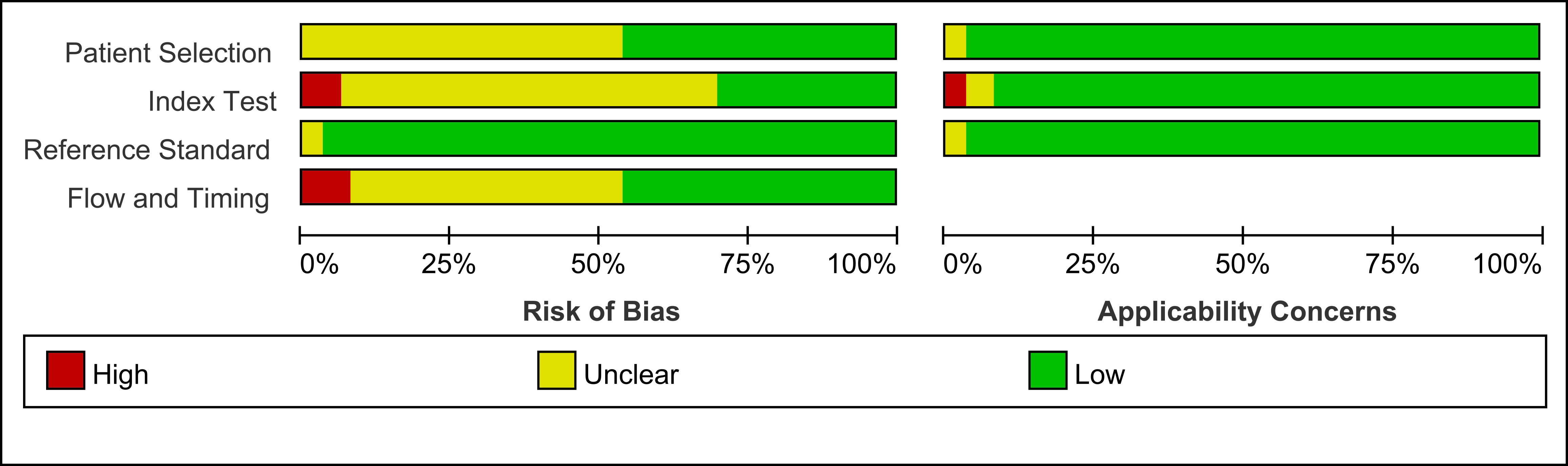
**

**Figure S1.** Methodological quality summary of studies included in the meta-analysis (63 studies).

**
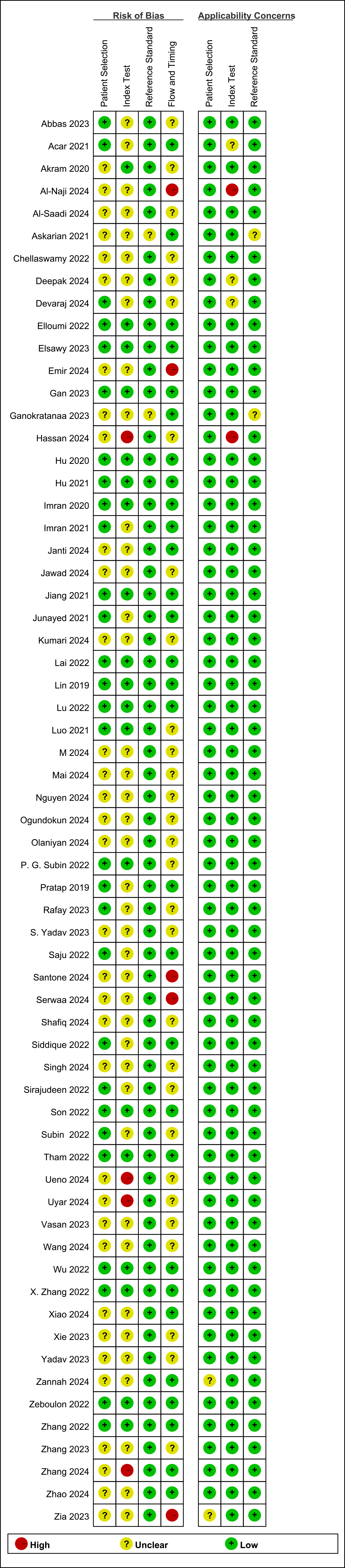
**

**Figure S2.** Methodological quality graph of studies included in the meta-analysis (63 studies).

**Appendix 1.** A summary of the search strategies used in each database, including specific search phrases, Boolean operations, field restrictions, etc.

Image Analysis-Based Deep Learning for Cataract Diagnosis: A Systematic Review and Meta-Analysis

Database：Pubmed、Cochrane、Embase、Web of science、IEEE

**1.** Cataracts

Lens Opacities

Lens Opacity

Opacities, Lens

Opacity, Lens

Cataract, Membranous

Cataracts, Membranous

Membranous Cataract

Membranous Cataracts

Pseudophakia

**2.** **Deep learning [Mesh]**

Machine learning

Artificial intelligence

Convolutional Neural Network

Neural network

Artificial neural network

Residual neural network

Graph neural network

Generative adversarial network

PubMed（517）

#1: ((((((((((Cataracts) OR (Lens Opacities)) OR (Lens Opacity)) OR (Opacities, Lens)) OR (Opacity, Lens)) OR (Cataract, Membranous)) OR (Cataracts, Membranous)) OR (Membranous Cataract)) OR (Membranous Cataracts)) OR (pseudophakia))

#2: ((((((((Deep learning) OR (Machine learning)) OR (Artificial intelligence)) OR (Convolutional Neural Network)) OR (Neural network)) OR (Artificial neural network)) OR (Residual neural network)) OR (Graph neural network)) OR (Generative adversarial network)

#3: #1 AND #2

Embase(145)

#40

'deep learning'/exp OR 'deep learning' OR (deep AND ('learning'/exp OR learning)) OR 'machine learning':ti,ab,kw OR 'artificial intelligence':ti,ab,kw OR 'convolutional neural network':ti,ab,kw OR 'neural network':ti,ab,kw OR 'artificial neural network':ti,ab,kw OR 'residual neural network':ti,ab,kw OR 'graph neural network':ti,ab,kw OR 'generative adversarial network':ti,ab,kw

#39

cataracts OR 'lens opacities':ti,ab,kw OR 'lens opacity':ti,ab,kw OR 'opacities, lens':ti,ab,kw OR 'opacity, lens':ti,ab,kw OR 'cataract, membranous':ti,ab,kw OR 'cataracts, membranous':ti,ab,kw OR 'membranous cataract':ti,ab,kw OR 'membranous cataracts':ti,ab,kw OR pseudophakia:ti,ab,kw

Cochrane (20)

#1: ((((((((((Cataracts) OR (Lens Opacities)) OR (Lens Opacity)) OR (Opacities, Lens)) OR (Opacity, Lens)) OR (Cataract, Membranous)) OR (Cataracts, Membranous)) OR (Membranous Cataract)) OR (Membranous Cataracts)) OR (pseudophakia))

#2: ((((((((Deep learning) OR (Machine learning)) OR (Artificial intelligence)) OR (Convolutional Neural Network)) OR (Neural network)) OR (Artificial neural network)) OR (Residual neural network)) OR (Graph neural network)) OR (Generative adversarial network)

#3: #1 AND #2

IEEE（704）

#1: ((((((((((Cataracts) OR (Lens Opacities)) OR (Lens Opacity)) OR (Opacities, Lens)) OR (Opacity, Lens)) OR (Cataract, Membranous)) OR (Cataracts, Membranous)) OR (Membranous Cataract)) OR (Membranous Cataracts)) OR (pseudophakia))

#2: ((((((((Deep learning) OR (Machine learning)) OR (Artificial intelligence)) OR (Convolutional Neural Network)) OR (Neural network)) OR (Artificial neural network)) OR (Residual neural network)) OR (Graph neural network)) OR (Generative adversarial network)

#3: #1 AND #2

Wos(849)

#1: Cataracts (Topic) or Lens Opacities (Topic) or Lens Opacity (Topic) or Opacities, Lens (Topic) or Opacity, Lens (Topic) or Cataract, Membranous (Topic) or Cataracts, Membranous (Topic) or Membranous Cataract (Topic) or Membranous Cataracts (Topic) or pseudophakia (Topic) and Preprint Citation Index (Exclude – Database)

#2: Deep learning (Topic) or Machine learning (Topic) or Artificial intelligence (Topic) or Convolutional Neural Network (Topic) or Neural network (Topic) or Artificial neural network (Topic) or Residual neural network (Topic) or Graph neural network (Topic) or Generative adversarial network (Topic) and Preprint Citation Index (Exclude – Database)

Total identified: 2,235

Duplicates removed: 492

Total records screened: 1,743
